# Modulation of the NLRP3 inflammasome by Sars-CoV-2 Envelope protein

**DOI:** 10.1038/s41598-021-04133-7

**Published:** 2021-12-24

**Authors:** Mustafa Yalcinkaya, Wenli Liu, Mohammad N. Islam, Andriana G. Kotini, Galina A. Gusarova, Trevor P. Fidler, Eirini P. Papapetrou, Jahar Bhattacharya, Nan Wang, Alan R. Tall

**Affiliations:** 1 Division of Molecular Medicine, Department of Medicine, Columbia University Irving Medical Center, New York, NY USA; 2 Lung Biology Lab, Division of Pulmonary, Allergy and Critical Care, Department of Medicine, Vagelos College of Physicians and Surgeons, Columbia University, New York, NY 10032 USA; 3 Department of Oncological Sciences, Icahn School of Medicine at Mount Sinai, New York, NY USA; 4 Tisch Cancer Institute, Icahn School of Medicine at Mount Sinai, New York, NY USA; 5 Black Family Stem Cell Institute, Icahn School of Medicine at Mount Sinai, New York, NY USA

**Keywords:** Immunology, Microbiology, Pathogenesis

## Abstract

Despite the initial success of some drugs and vaccines targeting COVID-19, understanding the mechanism underlying SARS-CoV-2 disease pathogenesis remains crucial for the development of further approaches to treatment. Some patients with severe Covid-19 experience a cytokine storm and display evidence of inflammasome activation leading to increased levels of IL-1β and IL-18; however, other reports have suggested reduced inflammatory responses to Sars-Cov-2. In this study we have examined the effects of the Sars-Cov-2 envelope (E) protein, a virulence factor in coronaviruses, on inflammasome activation and pulmonary inflammation. In cultured macrophages the E protein suppressed inflammasome priming and NLRP3 inflammasome activation. Similarly, in mice transfected with E protein and treated with poly(I:C) to simulate the effects of viral RNA, the E protein, in an NLRP3-dependent fashion, reduced expression of pro-IL-1β, levels of IL-1β and IL-18 in broncho-alveolar lavage fluid, and macrophage infiltration in the lung. To simulate the effects of more advanced infection, macrophages were treated with both LPS and poly(I:C). In this setting the E protein increased NLRP3 inflammasome activation in both murine and human macrophages. Thus, the Sars-Cov-2 E protein may initially suppress the host NLRP3 inflammasome response to viral RNA while potentially increasing NLRP3 inflammasome responses in the later stages of infection. Targeting the Sars-Cov-2 E protein especially in the early stages of infection may represent a novel approach to Covid-19 therapy.

## Introduction

Severe acute respiratory syndrome coronavirus (Sars-Cov)-2 infection is characterized by a strong inflammatory response, which is thought to promote organ damage and death^1^. Several studies have indicated that NLRP3 inflammasome activation may play a central role in this excessive inflammatory response^2^. Markers of NLRP3 inflammasome activation correlate with severity of disease in Covid-19 patients^3^. In mice multiple cytokines including those produced downstream of inflammasome activation induce hyper-inflammation and death in a combinatorial fashion during Cov-2 infection^4^. Moreover, some common conditions that are associated with increased inflammasome activation such as clonal hematopoiesis (CH) may be associated with a worse outcome in Covid-19 patients^5–7^. However, some reports have found a decreased pulmonary inflammatory response in early Covid-19 infection compared to other viral upper respiratory infections^8^ while others have found reduced plasma cytokine levels in Covid-19 patients with ARDS compared to other patients with ARDS^9^.

The NLRP3 inflammasome is a multiprotein complex that mediates the cleavage and activation of caspase-1, leading to cleavage of Gasdermin D and formation of membrane pores that permit the secretion of IL-1β, IL-18 and LDH from macrophages^10,11^. Inflammasome activation involves an initial priming step that leads to increased expression of inflammasome components followed by an activation step that may be mediated by a variety of cellular factors^12–14^. Infection by different RNA viruses can lead to NLRP3 inflammasome activation, which favors the host by aiding in viral clearance^15,16^. These responses depend on NLRP3 inflammasome activation in response to viral RNA and can be simulated by administration of poly(I:C)^15^.

The Envelope protein (E-protein) of coronaviruses is an enigmatic small protein, which can oligomerize to form a membrane ion-channel known as a viroporin^17,18^. Recent studies have indicated that the E protein has multiple functions during infection including in viral assembly and egress and in modulating the host stress response^19^. Studies in mice using Sars-Cov deficient in the E protein have shown that the E protein increases viral virulence^20^. The E protein localizes to the endoplasmic reticulum Golgi intermediate compartment (ERGIC) where it is thought to be involved in viral assembly and budding^21,22^. In cell culture, the Sars-Cov E-protein suppresses the cellular integrated stress response^23^, which could lead to suppression of inflammasome priming^24^. In the present study, we aimed to determine whether the Sars-Cov-2 E-protein, which is about 80% homologous to Sars-Cov E protein, regulates the NLRP3 inflammasome. We show in cell culture that the Sars-Cov-2 E protein suppresses the unfolded protein response (UPR) and NLRP3 inflammasome priming, leading to reduced NLRP3-dependent inflammatory responses. Moreover, using poly(I:C) to simulate the effects of viral RNA in vivo the E protein reduces the NLRP3 inflammatory response and decreases lung inflammation.

## Results

### Sars-Cov-2 Envelope protein decreases ER stress and inflammasome priming

**Figure 1:**
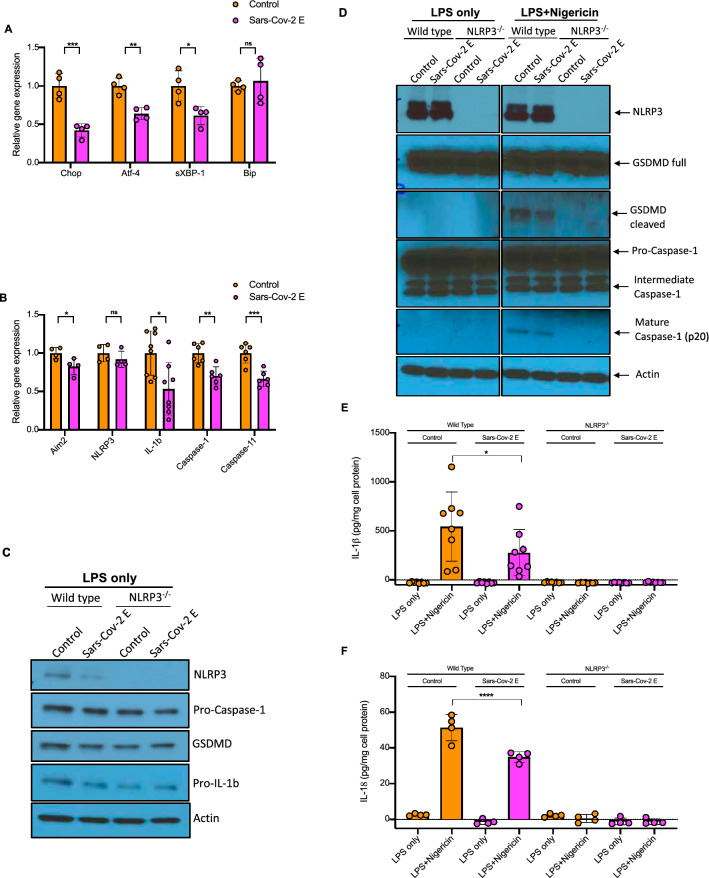
Sars-Cov-2 E-protein suppresses the ER stress response, inflammasome priming and activation. **(A)** Quantitative real-time PCR analysis of ER stress genes in bone marrow derived macrophages (BMDMs). Data are presented as mean ± SD, which were analyzed by unpaired t-test. ***P ≤ 0.001, **P ≤ 0.01, *P ≤ 0.05. **(B)** Quantitative real-time PCR analysis of inflammasome genes in BMDMs. Data are presented as mean ± SD, which were analyzed by unpaired t-test. ***P ≤ 0.001, **P ≤ 0.01, *P ≤ 0.05. **(C)** Western blot analysis of inflammasome proteins in cellular lysates of Wild type (WT) and *Nlrp3*^*−/−*^ BMDMs, transduced with control and E-protein lentiviruses for 72 h and were primed with 20 ng/ml LPS for 4 h. Blots were cut to probe with NLRP3 (from ~ 80 kDa to top) or pro-caspase-1, GSDMD, pro-IL-1β and Actin (from ~ 80 kDa to bottom). **(D)** Western blot analysis of inflammasome proteins in cellular lysates of WT and *Nlrp3*^*−/−*^ BMDMs, transduced with control and E-protein lentiviruses for 72 h and were primed with 20 ng/ml LPS for 3 h and treated with Nigericin for 1 h. **(E)** Quantification of IL-1β secretion into cell media in WT and *Nlrp3*^*−/−*^ BMDMs. BMDMs were transduced with control and E-protein lentiviruses for 72 h and then primed with 20 ng/ml LPS for 3 h and treated with Nigericin for 1 h. IL-1β secretion was assessed via IL-1β ELISA kit. Data are presented as mean ± SD, which were analyzed by one-way ANOVA coupled with Tukey’s test for multiple comparisons. ***P ≤ 0.001, **P ≤ 0.01, *P ≤ 0.05. **(F)** Quantification of IL-18 secretion into cell media in WT and *Nlrp3*^*−/−*^ BMDMs. BMDMs were transduced with control and E-protein lentiviruses for 72 h and then primed with 20 ng/ml LPS for 3 h and treated with Nigericin for 1 h. IL-18 secretion was assessed via IL-18 ELISA kit. Data are presented as mean ± SD, which were analyzed by one-way ANOVA coupled with Tukey’s test for multiple comparisons. ***P ≤ 0.001, **P ≤ 0.01, *P ≤ 0.05.

To assess the role of Sars-Cov-2 E-protein, bone marrow derived macrophages (BMDMs) were transduced with control and E-protein lentiviruses for 72 h at high efficiency (about 50%) (Supplementary Fig. S1A). Expression of E-protein in macrophages reduced ER stress markers such as *Chop* and *Atf4* expression as well as spliced *Xbp1* (Fig. 1A). Moreover, expression of inflammasome genes such as *Il1b*, *Caspase1*, *Caspase11* and *Aim2* was reduced by the E protein in unprimed cells (Fig. 1B). Moreover, expression of E-protein decreased NLRP3 and pro-IL-1β but not pro-caspase-1 and gasdermin D (GSDMD) protein levels in LPS primed BMDMs (Fig. 1C). To determine whether the E-protein regulates inflammasome activation, wild type (WT) and *Nlrp3*^*−/−*^ BMDMs were primed with 20 ng/ml Lipopolysaccharide (LPS) and treated with 10 μg/ml Nigericin, a K^+^ ionophore that activates the NLRP3 inflammasome. GSDMD and to lesser extent caspase-1 cleavage (Fig. 1D), IL-1β secretion (Fig. 1E) and IL-18 secretion (Fig. 1F) were reduced by E-protein in response to Nigericin treatment, indicating suppression of inflammasome activation. The E-protein suppressed secretion of TNF-α (Supplementary Fig. S1B) and IL-6 (Supplementary Fig. S1C in LPS primed BMDMs, consistent with suppression of inflammasome priming. The overexpression of E-protein had no effect on AIM2 inflammasome, measured by LDH release (Supplementary Fig. S1D) or IL-1β secretion (Supplementary Fig. S1E). These findings confirm that like the homologous protein in Sars-Cov, the Sars Cov-2 E protein suppresses the ER stress response and NLRP3 inflammasome activation.

### E-protein reduces NLRP3 inflammasome activation and inflammation in the lung

**Figure 2:**
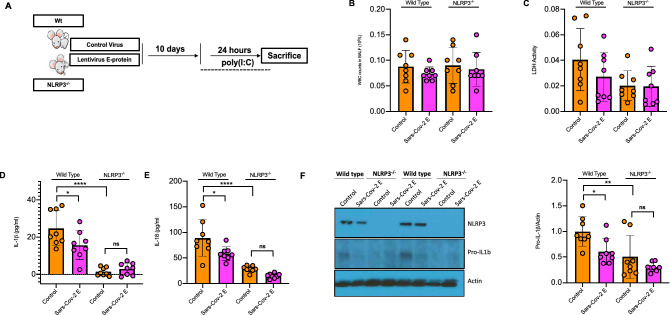
E-protein suppresses NLRP3 inflammasome activation in bronchoalveolar lavage fluid (BALF). **(A)** Schematic representation of poly(I:C). WT and *Nlrp3*^*−/−*^ mice were injected intranasally with control and E-protein lentiviruses for 10 days. The mice were challenged intranasally with poly(I:C) for 24 h and bronchoalveolar fluid (BALF) and lungs were harvested to assess inflammasome activation. **(B)** Total white blood cells (WBCs) in BALF. WBCs were analyzed with Hematology Analyzer. Data are presented as mean ± SD, which were analyzed by one-way ANOVA coupled with Tukey’s test for multiple comparisons. **(C)** Quantification of LDH release into BALF. LDH release was assessed via LDH kit. Data are presented as mean ± SD, which were analyzed by one-way ANOVA coupled with Tukey’s test for multiple comparisons. **(D)** Quantification of IL-1β secretion into BALF. IL-1β secretion was assessed via IL-1β ELISA. Data are presented as mean ± SD, which were analyzed by one-way ANOVA coupled with Tukey’s test for multiple comparisons. ****P ≤ 0.0001, ***P ≤ 0.001, **P ≤ 0.01, *P ≤ 0.05. **(E)** Quantification of IL-18 secretion into BALF. IL-18 secretion was assessed via IL-18 ELISA. Data are presented as mean ± SD, which were analyzed by one-way ANOVA coupled with Tukey’s test for multiple comparisons. ****P ≤ 0.0001, ***P ≤ 0.001, **P ≤ 0.01, *P ≤ 0.05. **(F)** Western blot analysis of pro-IL-1β and NLRP3 in cellular lysates of WBCs in BALF. Data are presented as mean ± SD, which were analyzed by one-way ANOVA coupled with Tukey’s test for multiple comparisons. Blots were cut to probe with NLRP3 (from ~ 80 kDa to top) or pro-IL-1β and Actin (from ~ 80 kDa to bottom).

To determine if the Sars-Cov-2 E protein would suppress the inflammatory response to viral RNA in vivo, , WT and *Nlrp3*^*−/−*^ mice were injected intranasally with control and E-protein lentiviruses for 10 days then challenged intranasally with poly (I:C) for 24 h (Fig. 2A) to simulate the effects of viral RNA^25,26^. The efficiency of transduction upon instillation was about 30% (Supplementary Fig. S2A). The total number of white blood cells (WBCs) and LDH levels in BAL fluid were not affected with either the E-protein or by *Nlrp3* deficiency (Fig. 2B,C). Similarly, TNFα, IL-6 and IFNβ levels were unchanged in BAL fluid (Supplementary Fig. S2B–D). However, IL-1β and IL-18 levels in BAL fluid were significantly decreased by E-protein (Fig. 2D–F). NLRP3 deficiency markedly reduced IL-1β and IL-18 levels and abrogated the effect of the E protein. Pro-IL-1β protein was reduced in BAL cells in E protein expressing mice, consistent with reduced inflammasome priming (Fig. 2F) and was further reduced by NLRP3 deficiency consistent with feed-forward priming by NLRP3 inflammasome activation. These findings indicate that the E protein reduces priming and activation of the NLRP3 inflammasome activation in response to poly(I:C) in the lung.

**Figure 3:**
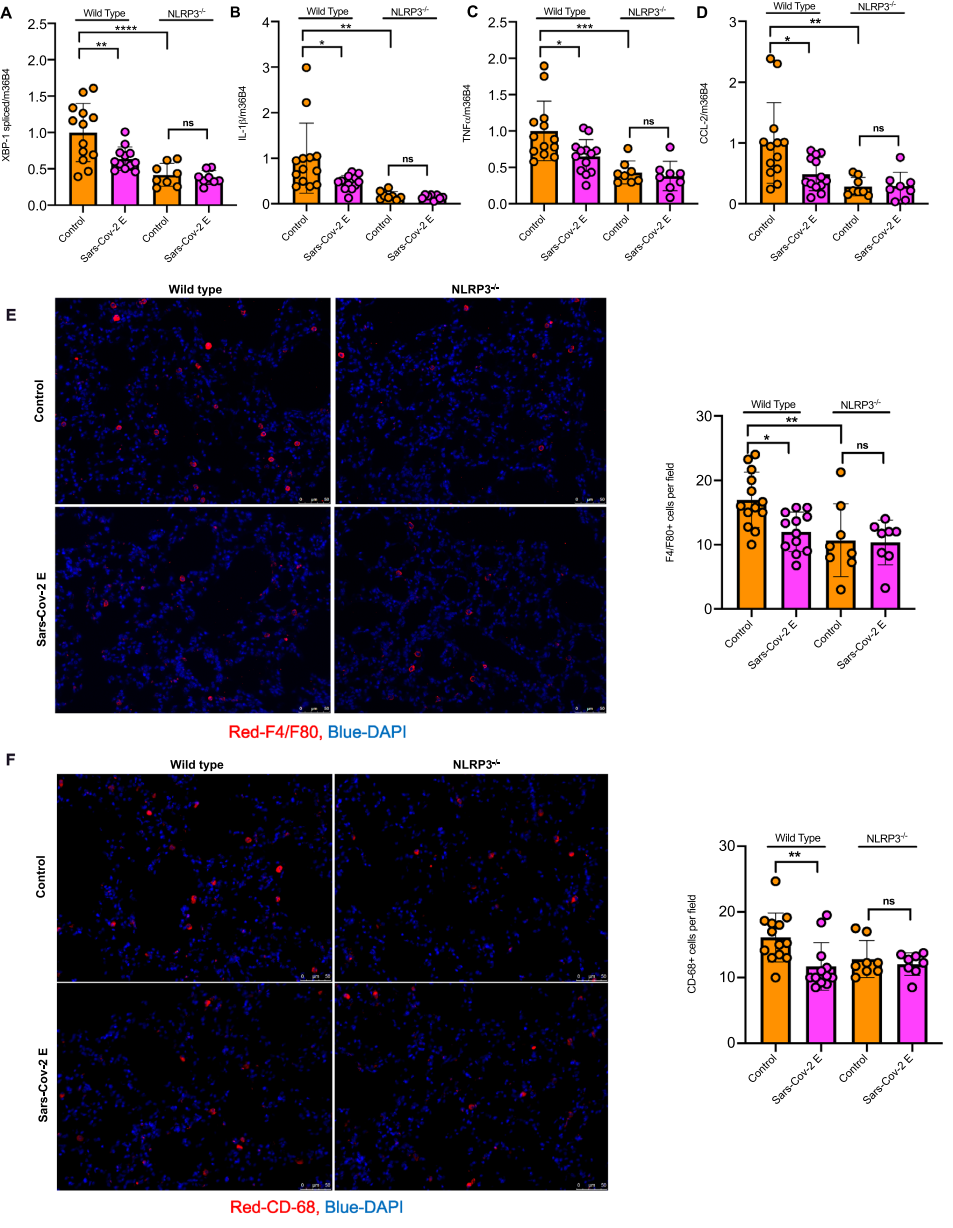
E-protein suppresses NLRP3 inflammasome priming and monocyte recruitment in the lung. **(A)** Quantitative real-time PCR analysis of spliced *Xbp-1* in whole lung of WT and *Nlrp3*^*−/−*^ mice, injected intranasally with control and E-protein lentiviruses for 10 days and challenged intranasally with poly (I:C) for 24 h. Data are presented as mean ± SD, which were analyzed by one-way ANOVA coupled with Tukey’s test for multiple comparisons. ****P ≤ 0.0001, ***P ≤ 0.001, **P ≤ 0.01, *P ≤ 0.05. **(B)** Quantitative real-time PCR analysis of *Il1b* in whole lung of WT and *Nlrp3*^*−/−*^ mice, injected intranasally with control and E-protein lentiviruses for 10 days and challenged intranasally with poly (I:C) for 24 h. Data are presented as mean ± SD, which were analyzed by one-way ANOVA coupled with Tukey’s test for multiple comparisons. ****P ≤ 0.0001, ***P ≤ 0.001, **P ≤ 0.01, *P ≤ 0.05. **(C)** Quantitative real-time PCR analysis of *Tnfa* in whole lung of WT and *Nlrp3*^*−/−*^ mice, injected intranasally with control and E-protein lentiviruses for 10 days and challenged intranasally with poly (I:C) for 24 h. Data are presented as mean ± SD, which were analyzed by one-way ANOVA coupled with Tukey’s test for multiple comparisons. ****P ≤ 0.0001, ***P ≤ 0.001, **P ≤ 0.01, *P ≤ 0.05. **(D)** Quantitative real-time PCR analysis of *Ccl2* in whole lung of WT and *Nlrp3*^*−/−*^ mice, injected intranasally with control and E-protein lentiviruses for 10 days and challenged intranasally with poly (I:C) for 24 h. Data are presented as mean ± SD, which were analyzed by one-way ANOVA coupled with Tukey’s test for multiple comparisons. ****P ≤ 0.0001, ***P ≤ 0.001, **P ≤ 0.01, *P ≤ 0.05. **(E)** Quantification of F4/80 + cells via immunostaining in whole lung of WT and *Nlrp3*^*−/−*^ mice, injected intranasally with control and E-protein lentiviruses for 10 days and challenged intranasally with poly (I:C) for 24 h. Data are presented as mean ± SD, which were analyzed by one-way ANOVA coupled with Tukey’s test for multiple comparisons. ****P ≤ 0.0001, ***P ≤ 0.001, **P ≤ 0.01, *P ≤ 0.05. **(F)** Quantification of CD-68 + cells via immunostaining in whole lung of WT and *Nlrp3*^*−/−*^ mice, injected intranasally with control and E-protein lentiviruses for 10 days and challenged intranasally with poly (I:C) for 24 h. Data are presented as mean ± SD, which were analyzed by one-way ANOVA coupled with Tukey’s test for multiple comparisons. ****P ≤ 0.0001, ***P ≤ 0.001, **P ≤ 0.01, *P ≤ 0.05.

We next analysed the inflammatory response in whole lung tissue. Real-time qPCR analyses showed that E-protein decreased ER stress marker *Xbp-1* splicing as well as expression of *Il1b*, *Tnfa* and *Ccl2* in WT but not *Nlrp3*^*−/−*^ mice (Fig. 3A–D). The expression of several other ER stress genes and cytokines was not significantly affected by E-protein (Supplementary Fig. S3A–J). To see whether immune cell infiltration into lungs is affected by E-protein, macrophages and neutrophil in lung sections were assessed by immunostaining with F4/F80 or CD-68 and S100A8, respectively. E-protein suppressed F4/F80 and CD-68 but not S100A8 positive cells in WT but not *Nlrp3*^*−/−*^ mice (Fig. 3E–F, Supplementary Fig. S3K). In conclusion, E-protein decreases NLRP3 inflammasome priming and activation and reduces pulmonary macrophage content, consistent with reduced inflammasome mediated monocyte recruitment.

### E-protein increases NLRP3 inflammasome response to poly(I:C) in LPS primed macrophages

**Figure 4:**
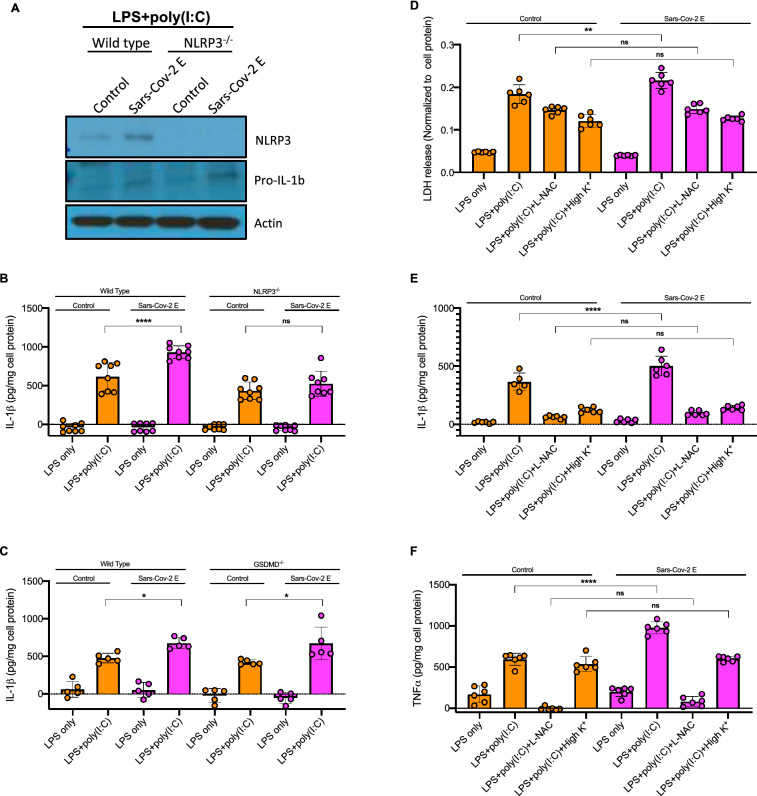
E-protein increases NLRP3 inflammasome response to poly(I:C), depending on K^+^ efflux and ROS but not GSDMD. **(A)** Western blot analysis of NLRP3 and pro-IL-1β protein levels in BMDMs, primed with 20 ng/ml LPS and treated with poly(I:C) for 16 h. Blots were cut to probe with NLRP3 (from ~ 80 kDa to top) or pro-IL-1β and Actin (from ~ 80 kDa to bottom). **(B)** Quantification of IL-1β secretion into cell media in Wild type (WT) and *Nlrp3*^*−/−*^ BMDMs. BMDMs were transduced with control and E-protein lentiviruses for 72 h and then primed with 20 ng/ml LPS for 3 h and treated with poly(I:C) for 16 h. IL-1β secretion was assessed via IL-1β ELISA kit. **(C)** Quantification of IL-1β secretion into cell media in WT and *Gsdmd*^*−/−*^ BMDMs. BMDMs were transduced with control and E-protein lentiviruses for 72 h and then primed with 20 ng/ml LPS for 3 h and treated with poly(I:C) for 16 h. IL-1β secretion was assessed via IL-1β ELISA kit. **(D)** Quantification of LDH release into cell media in BMDMs, transduced with control and E-protein lentiviruses for 72 h and then primed with 20 ng/ml LPS for 3 h and treated with poly(I:C) for 16 h. For inhibitor experiments, LPS-primed BMDM were preincubated with L-NAC (30 mM) 1 h prior to stimulation with poly(I:C). High K^+^ was supplied by adding 70 mM of K^+^ to extracellular media during stimulation with poly(I:C). LDH release was assessed via LDH kit. **(E)** Quantification of IL-1β secretion into cell media in BMDMs, transduced with control and E-protein lentiviruses for 72 h and then primed with 20 ng/ml LPS for 3 h and treated with poly(I:C) for 16 h. Same treatment was done as described in D. IL-1β secretion was assessed via IL-1β ELISA. **(F)** Quantification of TNF-α secretion into cell media in BMDMs, transduced with control and E-protein lentiviruses for 72 h and then primed with 20 ng/ml LPS for 3 h and treated with poly(I:C) for 16 h. Same treatment was done as described in D. TNF-α secretion was assessed via TNF-α ELISA. Data are presented as mean ± SD, which were analyzed by one-way ANOVA coupled with Tukey’s test for multiple comparisons. ***P ≤ 0.001, **P ≤ 0.01, *P ≤ 0.05.

Beyond the initial responses to viral RNA infection there may be further amplification of inflammatory responses by secondary bacterial infections or by pattern associated molecular patterns in damaged tissues that lead to activation of TLR4 signalling. Thus, we next assessed the role of the E-protein on NLRP3 inflammasome activation in response to both cytosolic poly(I:C), which has been employed to simulate the effects of dsRNA generated during viral replication^25–27^, and LPS treatment. BMDMs were primed with LPS and transfected with poly(I:C) via Lipofectamine 2000 for 16 h. In contrast to non-primed macrophages (Fig. 1B) or macrophages primed with LPS for 4 h (Fig. 1C), *Il1b* expression was not reduced by E-protein in longer LPS + poly(I:C) treated macrophages (Supplementary Fig. S4A). E-protein elevated NLRP3 and pro-IL-1β protein levels in response to LPS + poly(I:C) (Fig. 4A). Moreover, the E-protein increased IL-1β release in response to cytosolic poly(I:C). The increase in IL-1β secretion was abolished in *Nlrp3*^*−/−*^ macrophages (Fig. 4B) but not in *Gsdmd*^*−/−*^ macrophages (Fig. 4C). Furthermore, the E-protein stimulated TNFα secretion and LDH release as well as IL-1β secretion and all of these effects were inhibited by ROS inhibitor *N*-acetyl-l-cysteine (NAC) or by supplementation of cell medium with 70 mM K^+^ to block K^+^ efflux (Fig. 4D–F). These findings indicate that the Sars-Cov-2 E-protein increases the activation of the NLRP3 inflammasome leading to IL-1β and LDH release in response to cytosolic poly(I:C), in a process that depends on K^+^ efflux and ROS production but not *Gsdmd*. Seeking a possible link between reduced NF-κB activation by the E protein and increased inflammasome activation and TNFα release, we found that expression of *Tnfaip3* (A20), which is activated by NF-κB and mediates important negative feedback on expression of inflammatory genes, was reduced by the E protein in LPS + poly(I:C) treated cells (Supplementary Fig. S4B). In addition, *Ddx58* (RIG-1) but not *Mavs* expression was elevated by E-protein in response to LPS + poly(I:C) (Supplementary Fig. S4C-D).

### E-protein mediated inflammasome regulation is increased in JAK^V617F^ human iPSC-derived macrophages

**Figure 5:**
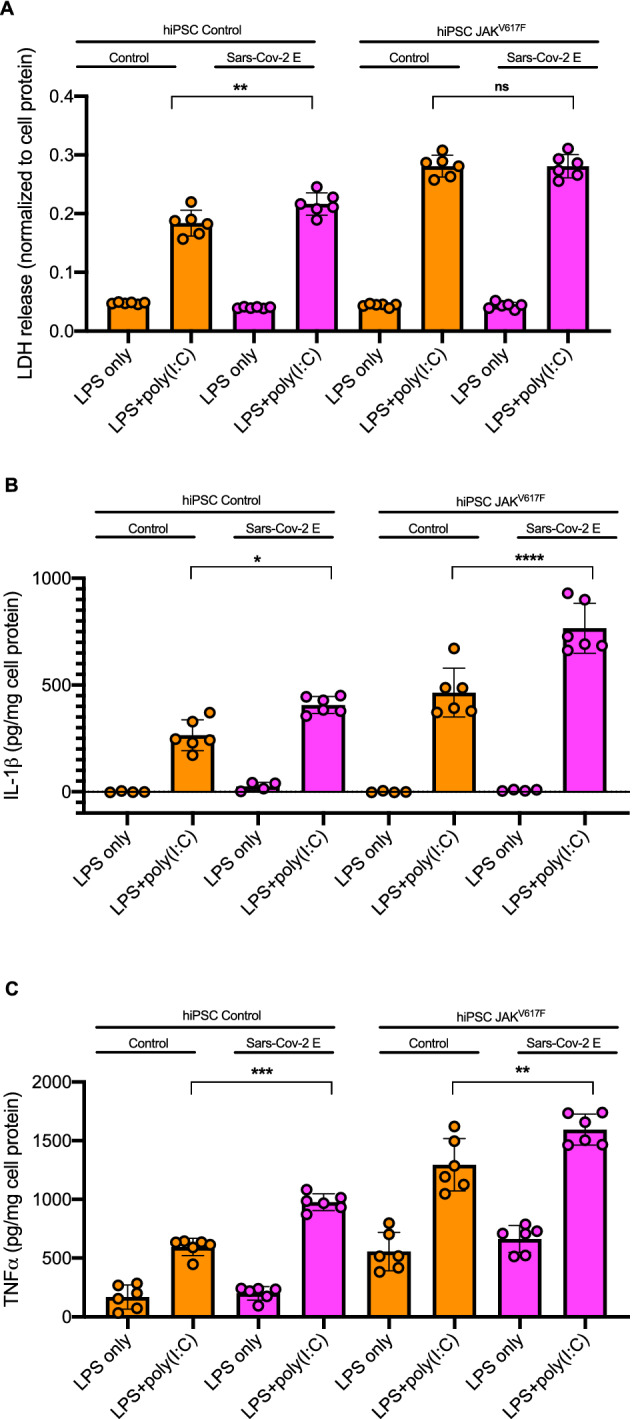
E-protein mediated inflammasome activation is more pronounced in JAK^V617F^ human iPSC-derived macrophages. **(A)** Quantification of LDH release into cell media in Control and JAK2^V617F^ hPSC-derived macrophages, transduced with control and E-protein lentiviruses for 72 h and then primed with 20 ng/ml LPS for 3 h and treated with poly(I:C) for 16 h. LDH release was assessed via LDH kit. Data are presented as mean ± SD, which were analyzed by one-way ANOVA coupled with Tukey’s test for multiple comparisons. ***P ≤ 0.001, **P ≤ 0.01, *P ≤ 0.05. **(B)** Quantification of IL-1β secretion into cell media in Control and JAK2^V617F^ hPSC-derived macrophages, transduced with control and E-protein lentiviruses for 72 h and then primed with 20 ng/ml LPS for 3 h and treated with poly(I:C) for 16 h. IL-1β secretion was assessed via IL-1β ELISA. Data are presented as mean ± SD, which were analyzed by one-way ANOVA coupled with Tukey’s test for multiple comparisons. ***P ≤ 0.001, **P ≤ 0.01, *P ≤ 0.05. **(C)** Quantification of TNF-α secretion into cell media in Control and JAK2^V617F^ hPSC-derived macrophages, transduced with control and E-protein lentiviruses for 72 h and then primed with 20 ng/ml LPS for 3 h and treated with poly(I:C) for 16 h. TNF-α secretion was assessed via TNF-α ELISA. Data are presented as mean ± SD, which were analyzed by one-way ANOVA coupled with Tukey’s test for multiple comparisons. ***P ≤ 0.001, **P ≤ 0.01, *P ≤ 0.05.

Recent studies have suggested that Sars-Cov-2 infection has more severe clinical consequences in subjects with underlying mutations in hematopoietic genes that cause clonal haematopoiesis^28^. To verify our findings in human macrophages and to assess the impact of underlying CH mutations on E protein modulation of inflammasome activation, we transduced human pluripotent stem cells (hPSC)-derived JAK2^V617F^, TET2 deficient and isogenic WT macrophages with lentivirus E protein and treated with inflammasome activators. When treated with E protein, WT macrophages showed reduced NLRP3 inflammasome activation in response to LPS + ATP (Supplementary Fig. S5A). When treated with poly(I:C) + LPS, the E protein promoted LDH and IL-1β release in WT macrophages (Fig. 5A,B). JAK2^V617F^ macrophages showed an increased release of LDH and IL-1β compared to isogenic WT control cells which in the case of IL-1β was further enhanced by the E protein. These effects were paralleled by increased Caspase-1 cleavage, that was more apparent in JAK2^V617F^ than WT macrophages (Supplementary Fig. S5B). Poly(I:C) treatment also caused an increased release of TNFα in WT macrophages that was further enhanced by the E protein, with both basal and E protein effects amplified in JAK2^V617F^ macrophages (Fig. 5C). Compared to WT isogenic controls, *TET2*^*−/−*^ macrophages showed increased IL-1β release in response to poly(I:C) consistent with data in mice showing increased NLRP3 inflammasome activation^29^ but this response was not further increased by Sars-Cov-2 E protein (Supplementary Fig. S5C). Treatment with increased K^+^ in medium or with NAC abrogated the effects of the E protein on IL-1β release in hPSC-derived macrophages (Supplementary Fig. S5D), similar to BMDMs. These findings indicate an increased inflammasome response to poly(I:C) in Sars-Cov-2 E protein expressing hPSC-derived macrophages that was further enhanced by the presence of one CH mutation (JAK2^V617F^) but not another (*TET2*^*−/−*)^.

## Discussion

Our studies suggest dual effects of Sars-Cov-2 E protein on inflammasome activation. On the one hand, the E protein suppresses inflammasome priming and reduces NLRP3 inflammasome activation in BMDMs or hPSC-derived macrophages. Most importantly, in mice treated with poly(I:C) to simulate the effects of viral RNA, the E protein suppresses inflammasome priming, NLRP3 inflammasome activation and inflammatory cell infiltration in the lung. This suggests that during the early stages of viral infection the E protein suppresses host NRLP3 inflammasome activation, which in addition to suppressing the unfolded protein response (UPR), may aid and protect viral replication. These findings resonate with recent observations showing decreased innate immune responses including decreased inflammasome priming as a major effect of early stage Covid-19^8^. On the other hand, when macrophages are primed with LPS and treated with poly(I:C) to mimic the effects of viral dsRNA, the E protein enhances NLRP3 inflammasome activation. This suggests that during later stages of infection when the inflammasome has been primed by LPS, DAMPS or other viral factors, the E protein in combination with viral RNA, may promote NLRP3 inflammasome activation. Thus, the E protein may mediate a biphasic effect during viral infection with initial immunosuppression followed by NLRP3 inflammasome activation in more advanced or complicated disease. Both effects may be detrimental to the host, suggesting that the E protein might represent a therapeutic target.

Previous studies have shown increased activation of the NLRP3 inflammasome by the SARS-Cov E protein, related to its viroporin ion channel activity. However, this occurred under conditions of overexpression of inflammasome components^30^ which would mask any effects of the E protein on priming. In contrast, our findings indicate that Sars-Cov-2 E protein suppresses the ER stress response, inflammasome priming and NLRP3 inflammasome activation. Several studies have demonstrated crosstalk between ER stress pathways and innate immune responses including the activation of NF-κB that can promote expression of inflammasome components such as *Il1b*^24,31,32^. In the setting of sustained and extreme ER stress especially during viral propagation, the activation of UPR and apoptotic pathways can be used by the host as an antiviral response^31,33^. Previous cell culture studies showed that Sars-Cov E protein could suppress the IRE-1/XBP-1 branch of UPR^23^. Our findings provide the first direct evidence that similar processes occur in the lung in response to the Sars-Cov-2 E protein and document that this leads to suppression of the NLRP3 inflammasome, reduced IL-1β and IL-18 and decreased infiltration of the lung with macrophages under conditions mimicking the effects of viral infection. This suggests that the E protein may suppress inflammasome priming and activation during early viral infection. Early suppression of the UPR and inflammasome priming may help the virus to escape early innate immune responses, that are likely beneficial to the host. Other studies have emphasized the role of viral factors that reduce Type 1 interferon responses^34–37^ but these were not observed in response to the E protein. The observations of high viral titers in the airways of the infected individuals with Covid-19^38,39^ may be explained by early innate immune suppression potentially involving several pathways including those mediated by the E protein.

Our findings showing immunosuppressive effects of the E protein are highly consistent with recent findings in early Covid-19 infection^8^ showing diminished innate immune responses compared to other upper respiratory viral infections. High throughput metagenomic sequencing and pathway analysis of cells obtained from the upper airway of patients with early Covid-19 infection showed a major suppression of inflammatory responses notably of genes mediating inflammasome priming and monocyte/macrophage recruitment. Interestingly, recent work has suggested inflammasome suppression by the Sars-Cov-2 nucleocapsid protein because of impaired GSDMD cleavage^40^. Together these studies strongly suggest an immunosuppressive effect of early Covid-19 infection, mediated in part by the viral E protein, that is permissive to continued viral replication leading to high viral titers increasing infectiousness and potential downstream adverse effects to the host.

Such downstream adverse events could include inflammasome activation as the Sars-Cov-2 E protein also enhanced NLRP3 inflammasome activation under specific conditions. This occurred in the setting of inflammasome priming by LPS followed by poly(I:C) treatment to simulate the effects of viral dsRNA. This suggests that the E protein may participate in later inflammasome activation in the host when the inflammasome has been primed by LPS, DAMPs or other viral components, and activated by viral RNA. This could perhaps explain pro-inflammatory effects of Sars-Cov E protein suggested in some earlier studies^20,30,41^. Several other Sars-Cov and Sars-Cov-2 structural proteins such as Orf3a, Orf8b and N-protein were previously reported to induce inflammasome activation^42–44^. In addition, a recent study has suggested that injected recombinant E protein can activate macrophage TLR2 to induce inflammatory gene expression^45^. This is not inconsistent with our findings since it may represent a more advanced stage of disease, compared to our model in which E protein was introduced from inside the cell, mimicking viral infection. Our study may reflect the early stages of the infection where viruses need to escape from immune system.

Activation of the NLRP3 inflammasome by poly(I:C) involves a pathway that appears distinct from classical NLRP3 inflammasome activation which leads to IL-1β and LDH release that is dependent of the pyroptosis-mediator GSDMD. The poly(I:C) activated pathway that is enhanced by the E protein depends on ROS and K^+^ efflux and appears similar to that described by Nunez and colleagues^25^. Double stranded viral RNA and poly(I:C) are sensed by the RIG-1 and MAVS proteins in macrophages activating type 1 interferon responses, TNFα secretion and other responses^46,47^. Franchi et al. showed that activation of the MAVS signaling pathway was upstream of NLRP3 inflammasome activation. E protein augmented the induction of *Ddx58* mRNA by poly(I:C), which correlates with the elevated NLRP3 inflammasome activation by E-protein response to poly(I:C). MAVS signaling also leads to TNF release which was also activated by E protein in our study. Kanneganti and co-workers have shown that initial impairment of NLRP3 inflammasome activation led to subsequent robust inflammatory cell death during coronavirus infection^48^. It is interesting to speculate that initial suppression of the UPR response by the E protein could be mechanistically tied to its subsequent enhancement of NLRP3 inflammasome responses. In this regard, it was of interest that there was repression of A20 by the E protein probably resulting from decreased NF-κB activation. Since A20 mediates negative feedback on TLR and MAVS responses^49,50^, this may condition macrophages to undergo an exaggerated response to TLR ligands. A recent study has shown that loss of feedback suppression of NF-κB signaling as would occur with repression of A20, dampens oscillatory NF-κB signaling and sets the stage for increased expression of inflammatory genes as a result of epigenetic reprogramming^51^. Further unraveling of these interconnected pathways could advance the understanding of Sars-Cov-2 pathogenesis.

Overall, our study provides preliminary data to suggest that the Sars-Cov-2 E protein suppresses NLRP3 inflammasome activation during the early stages of infection while in the later stages it may enhance NLRP3 inflammasome activation. Sars-Cov lacking E protein has been successfully used in animals as an attenuated viral vaccine that protects against subsequent viral infection^52^. The current focus on immunization with the Sars-Cov-2 spike protein, although partially successful, may be countered in part by viral mutations in the spike protein. Alternative therapeutic strategies could involve small molecules or vaccines targeting the Sars-Cov-2 E protein.

## Methods

### Mice

Wild-type C57BL/6 J ((The Jackson Laboratory, (000664)) and *Nlrp3*^*−/−*^ (The Jackson Laboratory, (B6.129S6-Nlrp3tm1Bhk/J) (21302)) were purchased from The Jackson Laboratory. All mice used for these studies were on a C57BL/6 J background and were housed in a specific pathogen-free facility under standard conditions of temperature (about 23 °C) with a 12-h light dark cycle and food available ad lib (humidity was not noted). Cages and water were changed every 14–21 days. All mouse experiments were approved by Institutional Animal Care and Use Committee of Columbia University and were conducted in accordance with the Institutional Animal Care and Use Committee of Columbia University guidelines.

### Mouse bone marrow derived macrophage (BMDM) culture

Bone marrow from Wild type or *Nlrp3*^*−/−*^ mice was flushed from hindlimbs with Hanks balanced salt solution (HBSS) and filtered in 60 μm cell filters on ice. Cells were centrifuged 1000 g for 10 min at 4 °C and suspended in DMEM with 10% FBS and 20% L-cell media (LCM), 100 U/ml Penicillin/Streptomycin (Thermo Fischer, 15,140,148). Bone marrow cells were then incubated in non-tissue culture treated flasks for 5 days and plated into new dishes overnight for the indicated assays.

### Human iPSC generation and macrophage differentiation

JAK2^V617F^ and isogenic JAK2 WT iPSCs were generated from peripheral blood mononuclear cells of a patient with primary myelofibrosis, obtained from the Hematological Malignancies Tissue Bank of Mount Sinai with informed consent under a protocol approved by a Mount Sinai Institutional Review Board, using Sendai virus reprogramming, as previously described^53^. All methods were carried out in accordance with Mount Sinai Institutional Review Board guidelines and regulations. iPSCs were cultured on mitotically inactivated MEFs with hESC media supplemented with 6 ng/ml FGF2, as described^53^. TET2^*−/−*^ and WT parental HUES8 hESCs were previously generated through CRISPR/Cas9 gene editing^54^. Human pluripotent stem cells (hPSCs, including iPSCs and ESCs) were cultured on mitotically inactivated MEFs with hESC media supplemented with 6 ng/ml FGF2, as described^53^. Hematopoietic lineage specification was performed following a previously described spin-embryoid body-based protocol to generate hematopoietic progenitor cells through a hemogenic endothelium intermediate^53^. On day 10, the cells were transferred to macrophage differentiation culture consisting of StemPro-34 SFM medium with 1% nonessential amino acids (NEAA), 1 mM l-glutamine and 0.1 mM β-mercaptoethanol (2ME), supplemented with 100 ng/ml macrophage colony-stimulating factor (M-CSF) and 25 ng/ml interleukin 3 (IL-3) for 11 days with media changes every two days.

### Viral transduction of BMDMs and hPSC-derived macrophages

Following differentiation, macrophages were incubated with 2.10^5^ TU/ml control (LP156-100, Genecopoiea) and E-protein lentiviruses (LPP-CoV224-Lv215, Genecopoiea) for 24 h in complete growth medium supplemented with 8 μg/ml polybrene (Sigma, TR-1003). 24 h later, cells were supplemented with fresh media for 2 additional days. Transduction efficiency was assessed by GFP-positive cells under fluorescent microscope.

### Inflammasome activation in BMDMs and hPSC-derived macrophages

Following transduction, macrophages were preincubated with LPS (20 ng/ml), for 3 h then incubated with 10 μg/ml Nigericin for 1 h or transfected with 1 μg/ml poly(dA:dT)(tlrl-patn, InvivoGen) or poly(I:C) (tlrl-pic-5, InvivoGen) via lipofectamine 2000 (Invivogen, Cat # tlrl-patn) for 6 h or 16 h. For inhibitor experiments, LPS-primed BMDMs or hPSC-derived macrophages were preincubated with NAC (30 mM) 1 h prior to stimulation with poly(I:C). K^+^ supplementation was done by adding 70 mM of K^+^ to extracellular media during stimulation with poly(I:C). At the end of the treatment, cytokines in the media were measured using ELISA kits. Media was taken from BMDMs or hPSC-derived macrophages upon indicated treatments and analyzed for LDH activity with CyQUANTTM LDH Cytotoxicity Assay (Thermo Fisher Scientific, C20301). Data were normalized to protein concentration of cell lysates.

### Intranasal lentivirus injection and poly (I:C) challenge

Animals were anesthetized and challenged by intranasal administration of 50 μl control and E-protein lentiviruses (10^7^ TU/ml) in PBS. 10 days later, wild-type and NLRP3^*−/−*^ mice received 50 μg of poly(I:C) for 24 h. Next day, the mice were anesthetized via an IP injection of a mixture of ketamine (200 mg/kg) and xylazine (10 mg/kg). A catheter was inserted into the trachea by way of an injected opening located in the cervical part, and the airways were washed three times with a total of 1 ml PBS. The total cells in bronchoalveolar lavage fluid (BALF) were quantified with FORCYTE Veterinary Hematology Analyzer (Oxford Science, Inc.) and BALF was centrifuged at 1000×*g* for 5 min at 4 °C. The supernatant was used to assess cytokine and LDH release and the cells were lysed to determine specific proteins.

### Real-time quantitive PCR

For gene expression analysis, BMDMs or one lung lobe was harvested into TRIazol. Total RNA was isolated with a RNeasy kit (Qiagen) and cDNA was generated using a cDNA synthesis kit (Thermo Fisher Scientific, K1671). qPCR was conducted for specific genes and normalized to m36B4. Primers for qPCR assays are as follows: *m36b4* (For: CCTGAAGTGCTCGACATCAC; Rev: CCACAGACAATGCCAGGAC), *Ccl2* (For: CCCAATGAGTAGGCTGGAGA; Rev: TCTGGACCCATTCCTTCTTG), *Ccl3* (For: ACTGCCTGCTGCTTCTCCTACA; Rev: AGGAAAATGACACCTGGCTGG), *Ccl4* (For: AAACCTAACCCCGAGCAACA; Rev: CCATTGGTGCTGAGAACCCT), *Il1b* (For: TGTGAATGCCACCTTTTGACA; Rev: GGTCAAAGGTTTGGAAGCAG), *Il18* (For: GACTCTTGCGTCAACTTCAAGG; Rev: CAGGCTGTCTTTTGTCAACGA), *Il6* (For: ACAACCACGGCCTTCCCTACTT; Rev: CACGATTTCCCAGAGAACATGTG), *Caspase-1* (For: GAGACATATAAGGGAGAACGC; Rev: ATGGCACACCACAGATATCGG), *Caspase-11* (For: ACAATTGCCACTGTCCAGGT; Rev: CATTGCTGACCTTATTTCTGTATGG), *Nlrp3* (For: ATTACCCGCCCGAGAAAGG; Rev: TCGCAGCAAAGATCCACACAG), *Aim2* (For: GATTCAAAGTGCAGGTGCGG; Rev: TCTGAGGCTTAGCTTGAGGAC), *Tnfa* (For: CCAGACCCTCACACTCAGATC; Rev: CACTTGGTGGTTTGCTACGAC), *Ifnb* (For: C TGAACTCCACCAGCAGACAG; Rev: AAGATCTCTGCTCGGACCAC), *Spliced Xbp1* (For: CTGAGTCCGAATCAGGTGCAG; Rev: GTCCATGGGAAGATGTTCTGG), *Atf4* (For: GGGTTCTGTCTTCCACTCCA; Rev: AAGCAGCAGAGTCAGGCTTCC), *Chop* (For: CCACCACACCTGAAAGCAGAA; Rev: AGGTGAAAGGCAGGGACTCA), *Bip* (For: TTCAGCCAATTATCAGCAAACTCT; Rev: TTTTCTGATGTATCCTCTTCACCAGT), *Mavs* (For: CTGCCTCACAGCTAGTGACC; Rev: CCGGCGCTGGAGATTATTG) and *Ddx58* (For: AAGAGCCAGAGTGTCAGAATCT; Rev: AGCTCCAGTTGGTAATTTCTTGG).

### Immunohistochemistry

Another lobe from the lung was embedded in paraffin and then serially sectioned. Paraffin-embedded slides were deparaffinized and rehydrated in Trilogy (Cell MARQUE 920P-09). Identification of macrophages and neutrophils were performed by immunostaining using anti-F4/F80 (#70076, Cell Signaling, 1:200) or anti-CD68 (#ab125212, Abcam, 1:200) and anti-S100A8 (NBP2-27067, Novus, 1:200), respectively. The sections were incubated with primary antibodies overnight at 4 °C then incubated with secondary antibodies for 30 min. Sections were mounted using ProLong Gold Antifade Mountant with DAPI (Thermofisher, P3693) and imaged using a Leica DMI6000B microscope.

### Western blotting

BMDMs or hiPCS macrophages were lysed in RIPA buffer containing protease inhibitor on the ice for 30 min and then centrifuged at 14,000×*g* for 5 min. Protein lysates were separated by 4–20% gradient SDS-PAGE and transferred to nitrocellulose membranes. Then the membranes were blocked with 5% non-fat milk in TBS-T and incubated with primary antibodies, anti-Caspase-1 (14-9832-82, eBioScience, 1:2000 for mouse and Cell signalling 3866 T, 1:1000 for human), anti-GSDMD (Genentech, 1:1000), anti-NLRP3 (15101S, Cell Signalling, 1:1000), anti-IL-1β (Cell signalling 12426S, 1:1000) and β-actin (cell signalling 4970S, 1:5000) at 4 °C overnight and detected using HRP-conjugated secondary antibodies.

### Statistical analysis

The data sets for all validation experiments were analyzed using the GraphPad Prism 8 software. Comparison between groups was performed using one-way ANOVA coupled with Tukey’s test for multiple comparisons. Values are expressed as mean ± SD. P < 0.05 was regarded as significant.

### Ethical statement

All experimental protocols were approved by the Institutional Biosafety Committee of Columbia University. All methods were carried out in accordance with Columbia University Guidelines and Regulations. The study is reported in accordance with ARRIVE guidelines.

## Supplementary Information

Supplementary Information.
