# Supplementary material for: Modulation of the NLRP3 inflammasome by Sars-CoV-2 Envelope protein
Source: Sci Rep. 2021 Dec 24;11:24432. doi: 10.1038/s41598-021-04133-7 (PMC8709866; doi:10.1038/s41598-021-04133-7)
Supplement: Supplementary file 1 — Supplementary Information. [file 41598_2021_4133_MOESM1_ESM.pdf]

Title: Modulation of the NLRP3 inflammasome by Sars-CoV-2 Envelope Protein

Authors: Mustafa Yalcinkaya[1], Wenli Liu[1], Mohammad N. Islam[2], Andriana G. Kotini[3-5], Galina A. Gusarova[2], Trevor Fidler[1], Eirini P. Papapetrou[3-5], Jahar Bhattacharya[2], Nan Wang[1], Alan R. Tall[1]*.

Supplementary Figure 1

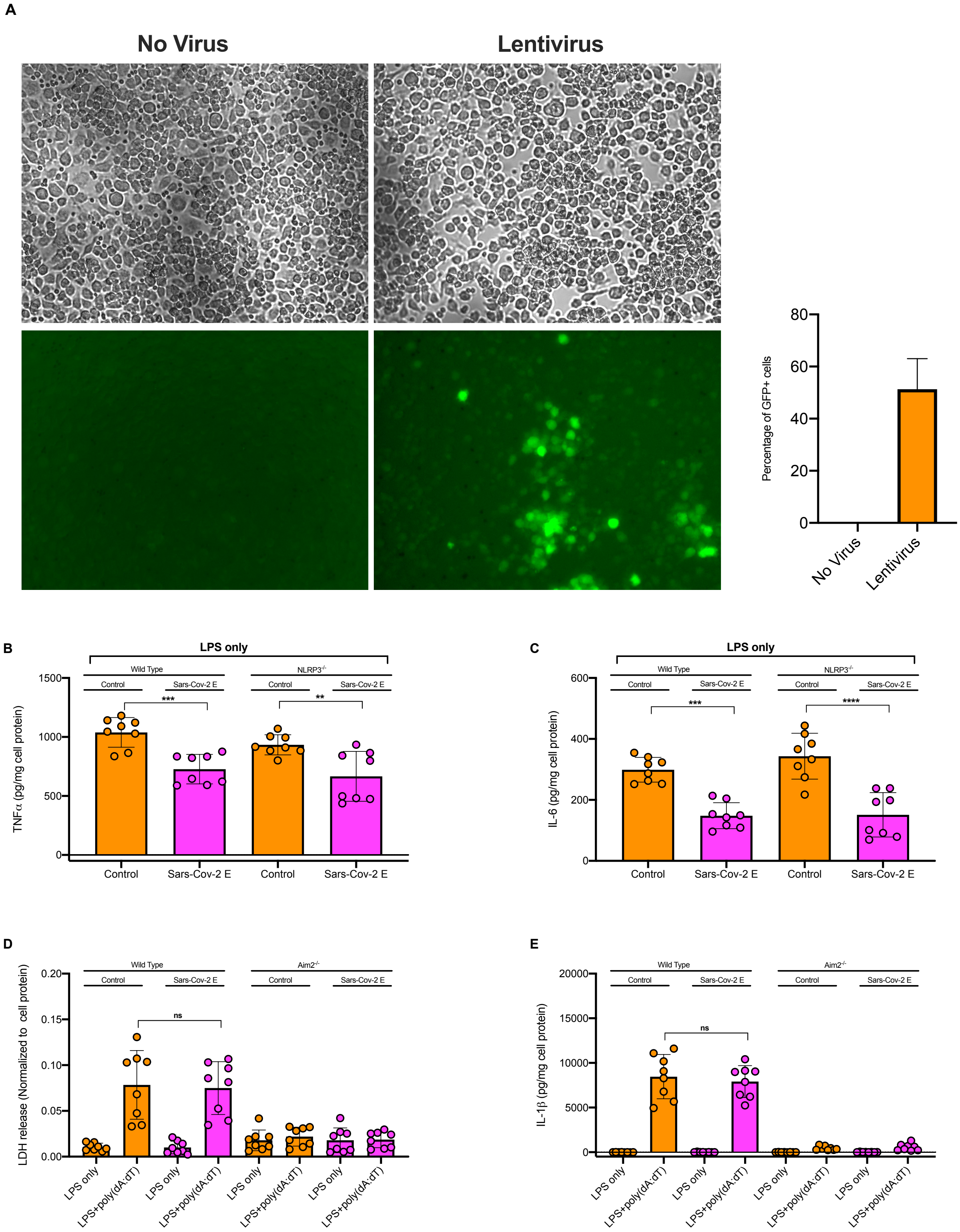

Supplementary Figure 1: E-protein suppresses inflammasome priming but does not affect Aim2 inflammasome response to poly(dA:dT).

(A) Bone marrow derived macrophages (BMDMs) were transduced with control and E-protein lentiviruses for 72h. Transduced cells (GFP positive) were analyzed by fluorescent microscope.

(B) Quantification of TNF-α secretion into cell media in Wild type (WT) and *Nlrp3*$^{-/-}$ BMDMs. BMDMs were transduced with control and E-protein lentiviruses for 72h and then primed with 20 ng/ml LPS for 4h. TNF-α secretion was assessed via TNF-α ELISA kit. Data are presented as mean ± SD, which were analyzed by one-way ANOVA coupled with Tukey's test for multiple comparisons.

(C) Quantification of IL-6 secretion into cell media in WT and *Nlrp3*$^{-/-}$ BMDMs. BMDMs were transduced with control and E-protein lentiviruses for 72h and then primed with 20 ng/ml LPS for 4h.

(D) Quantification of LDH release into cell media in BMDMs, transduced with control and E-protein lentiviruses for 72h and then primed with 20 ng/ml LPS for 3h and treated with poly(dA:dT) for 6h.

(E) Quantification of IL-1β secretion into cell media in BMDMs, transduced with control and E-protein lentiviruses for 72h and then primed with 20 ng/ml LPS for 3h and treated with poly(dA:dT) for 6h. ***$P \leq 0.001$, **$P \leq 0.01$, *$P \leq 0.05$

Supplementary Figure 2

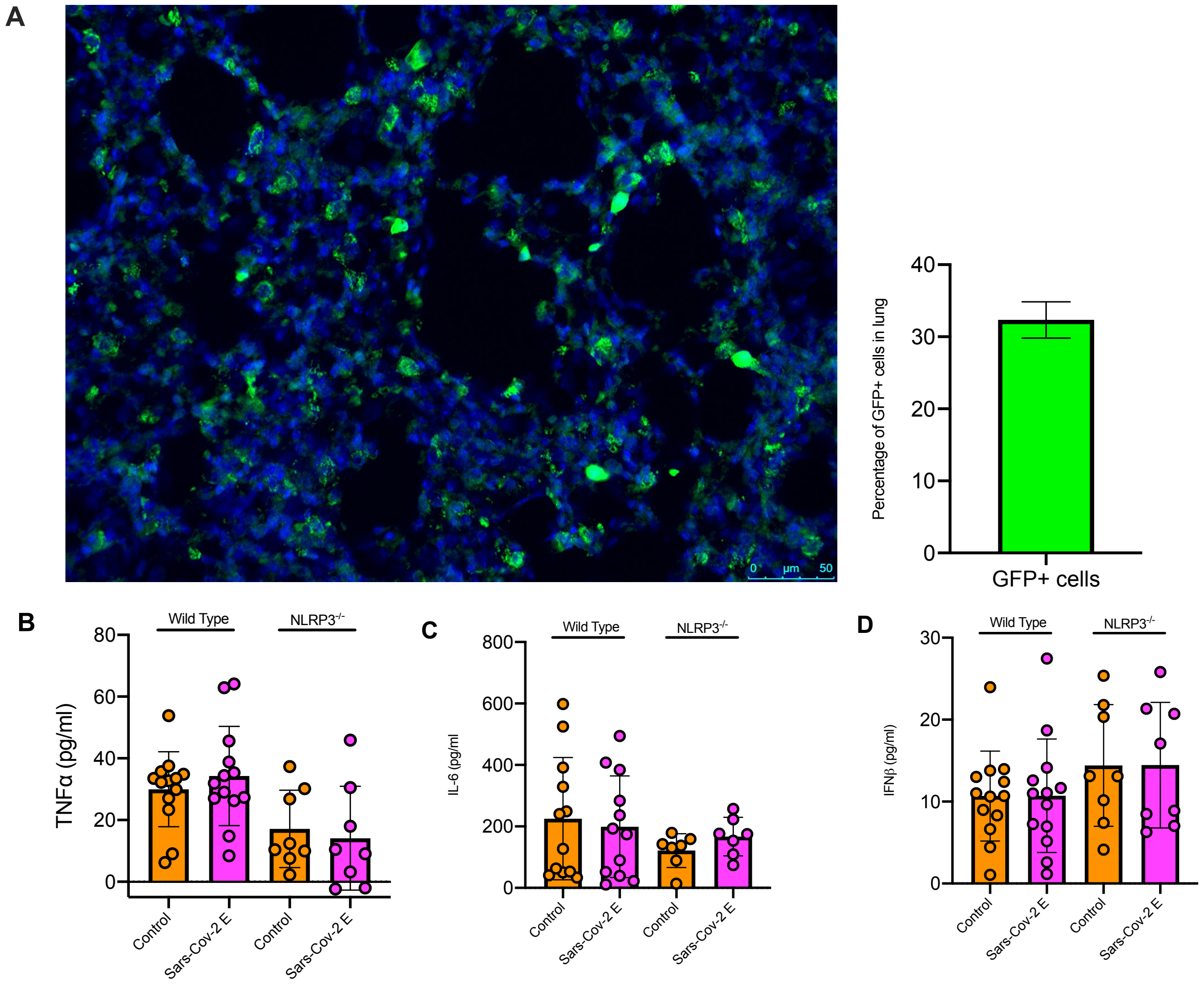

Supplementary Figure 2: E-protein did not have an effect on other cytokines in bronchoalveolar lavage fluid (BALF).

(A) Quantification of GFP+ cells in lung sections. Transduced cells (GFP positive) were analyzed by fluorescent microscope.

(B) Quantification of TNF-α secretion into BALF. TNF-α secretion was assessed via TNF-α ELISA. Data are presented as mean ± SD, which were analyzed by one-way ANOVA coupled with Tukey's test for multiple comparisons. ****P≤ 0.0001, ***P≤ 0.001, **P≤ 0.01, *P≤ 0.05

(C) Quantification of IL-6 secretion into BALF. IL-6 secretion was assessed via IL-6 ELISA. Data are presented as mean ± SD, which were analyzed by one-way ANOVA coupled with Tukey's test for multiple comparisons. ****P≤ 0.0001, ***P≤ 0.001, **P≤ 0.01, *P≤ 0.05

(D) Quantification of IFN-β secretion into BALF. IFN-β secretion was assessed via IFN-β ELISA. Data are presented as mean ± SD, which were analyzed by one-way ANOVA coupled with Tukey's test for multiple comparisons. ****P≤ 0.0001, ***P≤ 0.001, **P≤ 0.01, *P≤ 0.05

Supplementary Figure 3

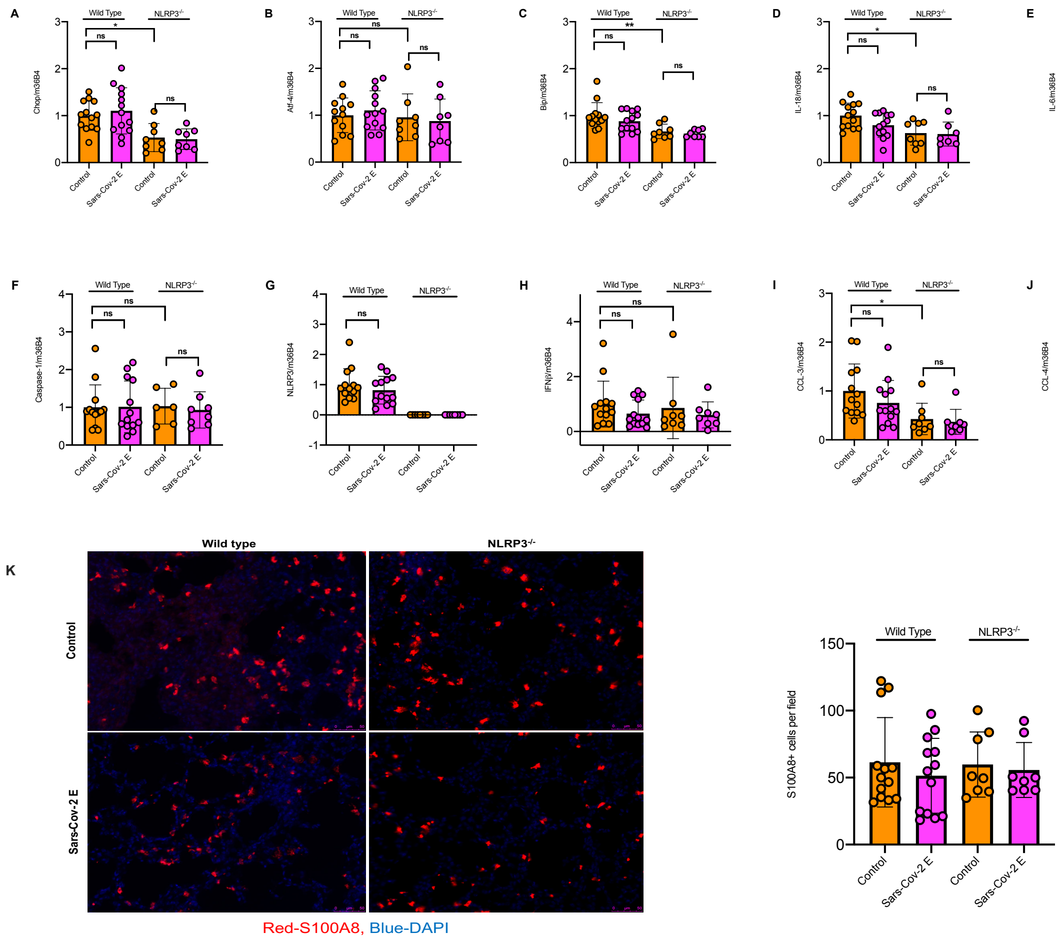

Supplementray Figure 3: E-protein did not affect neutrophil recruitment.

(A) Quantitative real-time PCR analysis of spliced *Chop* in whole lung of Wt and Nlrp3$^{-/-}$ mice.

(B) Quantitative real-time PCR analysis of *Atf4* in whole lung of Wt and Nlrp3$^{-/-}$ mice.

(C) Quantitative real-time PCR analysis of *Bip* in whole lung of Wt and Nlrp3$^{-/-}$ mice.

(D) Quantitative real-time PCR analysis of *Il18* in whole lung of Wt and Nlrp3$^{-/-}$ mice.

(E) Quantitative real-time PCR analysis of *Il6* in whole lung of Wt and Nlrp3$^{-/-}$ mice.

(F) Quantitative real-time PCR analysis of *Caspase1* in whole lung of Wt and Nlrp3$^{-/-}$ mice.

(G) Quantitative real-time PCR analysis of *Nlrp3* in whole lung of Wt and Nlrp3$^{-/-}$ mice.

(H) Quantitative real-time PCR analysis of *Ifnb* in whole lung of Wt and Nlrp3$^{-/-}$ mice.

(I) Quantitative real-time PCR analysis of *Ccl3* in whole lung of Wt and Nlrp3$^{-/-}$ mice.

(J) Quantitative real-time PCR analysis of *Ccl4* in whole lung of Wt and Nlrp3$^{-/-}$ mice.

(K) Quantification of S100A8+cells via immunostaining in whole lung of WT and *Nlrp3*$^{-/-}$ mice, injected intranasally with control and E-protein lentiviruses for 10 days and challenged intranasally with poly (I:C) for 24h. Data are presented as mean ± SD, which were analyzed by one-way ANOVA coupled with Tukey's test for multiple comparisons. ****P≤ 0.0001, ***P≤ 0.001, **P≤ 0.01, *P≤ 0.05

Supplementary Figure 4

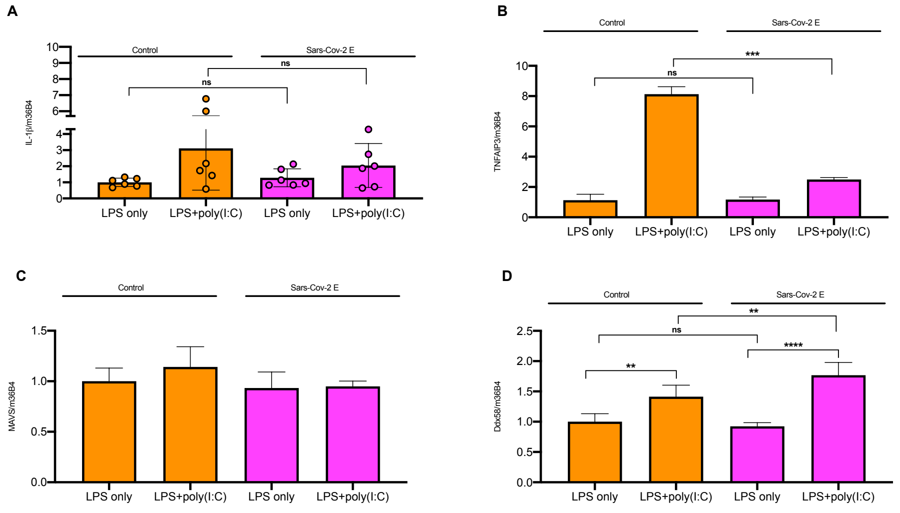

Supplementary Figure 4: E-protein decreases *Tnfaip3* (A20) and increases Ddx58 (RIG-1) expression response to poly(I:C), respectively.

(A) Quantitative real-time PCR analysis of *Il1b* gene expression in BMDMs, primed with 20 ng/ml LPS and treated with poly(I:C) for 16h.Data are presented as mean ± SD, which were analyzed by unpaired t-test. ***P≤ 0.001, **P≤ 0.01, *P≤ 0.05

(B) Quantitative real-time PCR analysis of *Tnfaip3* (A20) gene expression in BMDMs, primed with 20 ng/ml LPS and treated with poly(I:C) for 16h.Data are presented as mean ± SD, which were analyzed by unpaired t-test. ***P≤ 0.001, **P≤ 0.01, *P≤ 0.05

(C) Quantitative real-time PCR analysis of *Mavs* gene expression in BMDMs, primed with 20 ng/ml LPS and treated with poly(I:C) for 16h.Data are presented as mean ± SD, which were analyzed by unpaired t-test. ***P≤ 0.001, **P≤ 0.01, *P≤ 0.05

(D) Quantitative real-time PCR analysis of *Ddx58* gene expression in BMDMs, primed with 20 ng/ml LPS and treated with poly(I:C) for 16h.Data are presented as mean ± SD, which were analyzed by unpaired t-test. ***P≤ 0.001, **P≤ 0.01, *P≤ 0.05

Supplementary Figure 5

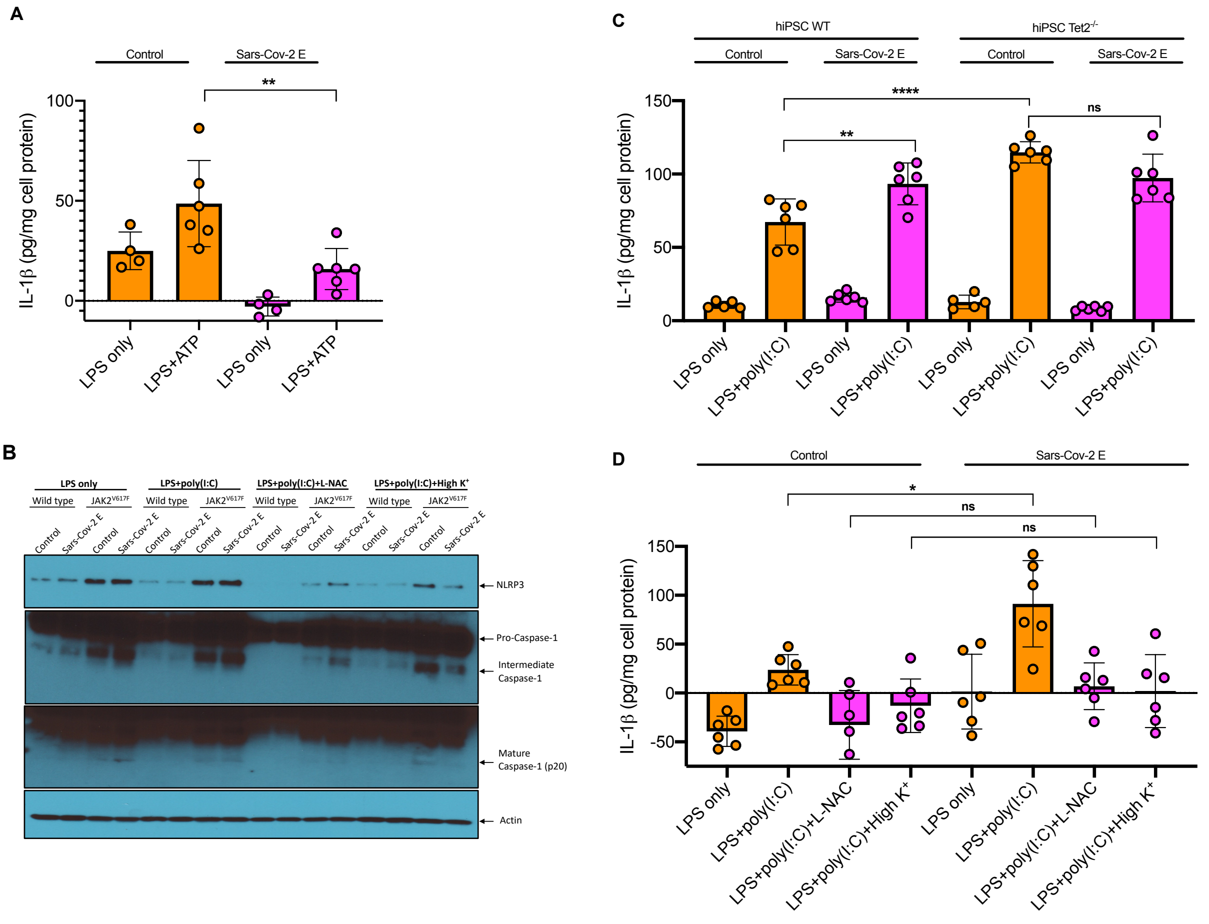

Supplementary Figure 5: E-protein mediated inflammasome regulation is more pronounced in JAK^{V617F} human but not TET2$^{-/-}$ hPSC-derived macrophages.

(A) Quantification of IL-1β secretion into cell media in WT hPSC-derived macrophages, transduced with control and E-protein lentiviruses for 72h and then primed with 20 ng/ml LPS for 3h and treated with ATP for 1h. IL-1β secretion was assessed via IL-1β ELISA. Data are presented as mean ± SD, which were analyzed by one-way ANOVA coupled with Tukey's test for multiple comparisons. ***P≤ 0.001, **P≤ 0.01, *P≤ 0.05

(B) Western blot analysis of inflammasome proteins in cellular lysates of WT and JAK2^{V617F} hPSC-derived macrophages, transduced with control and E-protein lentiviruses for 72h and then primed with 20 ng/ml LPS for 3h and treated with poly(I:C) for 16h.

(C) Quantification of IL-1β secretion into cell media in WT and TET2$^{-/-}$ hPSC-derived macrophages, transduced with control and E-protein lentiviruses for 72h and then primed with 20 ng/ml LPS for 3h and treated with poly(I:C) for 16h. IL-1β secretion was assessed via IL-1β ELISA.

(D) Quantification of IL-1β secretion into cell media in hPSC-derived macrophages, transduced with control and E-protein lentiviruses for 72h and then primed with 20 ng/ml LPS for 3h and treated with poly(I:C) for 16h. For inhibitor experiments, LPS-primed hPSC-derived macrophages were preincubated with L-NAC (30 mM) 1h prior to stimulation with poly(I:C). High K^+ was supplied by adding 70 mM of K^+ to extracellular media during stimulation with poly(I:C). IL-1β secretion was assessed via IL-1β ELISA. Data are presented as mean ± SD, which were analyzed by one-way ANOVA coupled with Tukey's test for multiple comparisons. ***P≤ 0.001, **P≤ 0.01, *P≤ 0.05

Source Data 1C

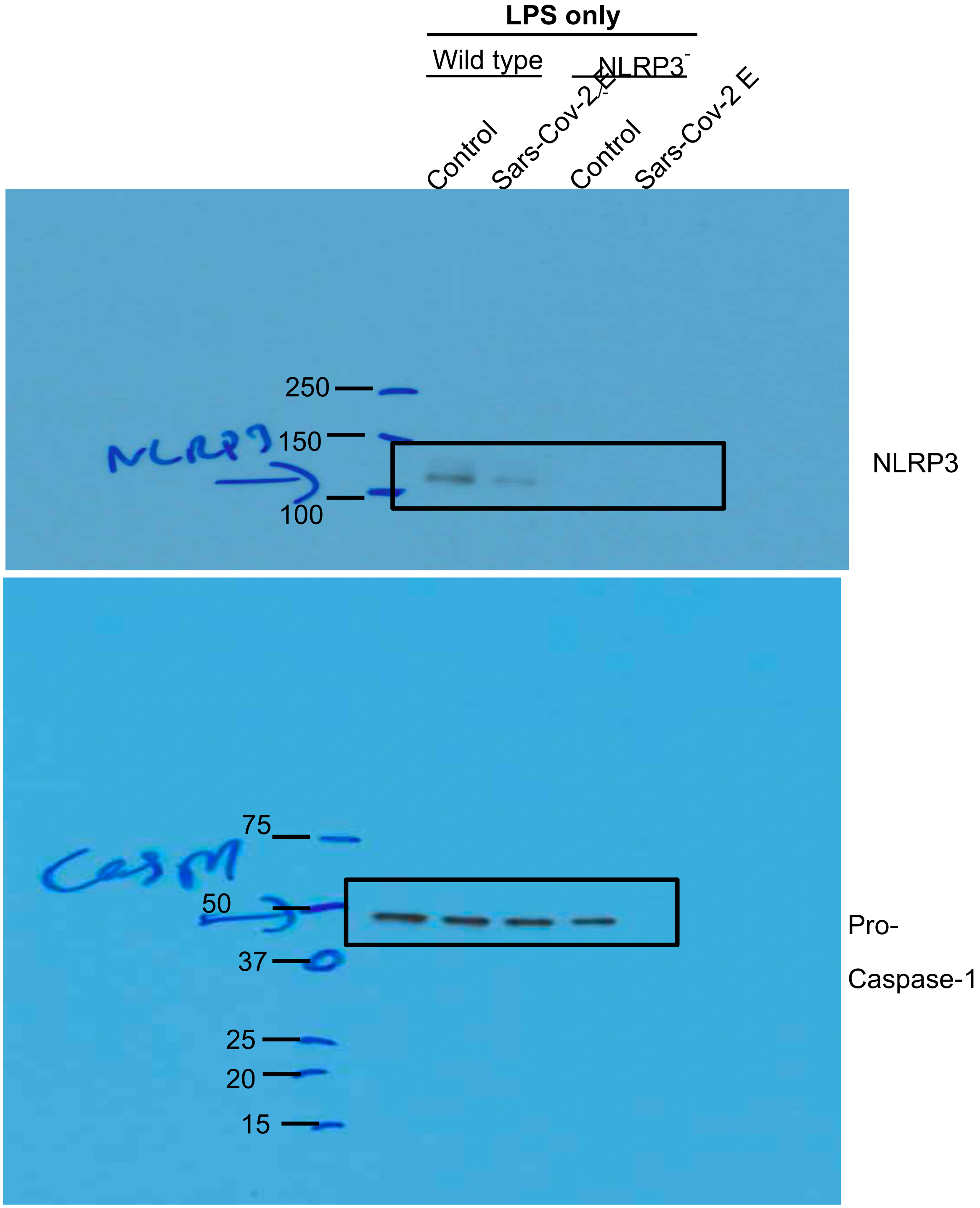

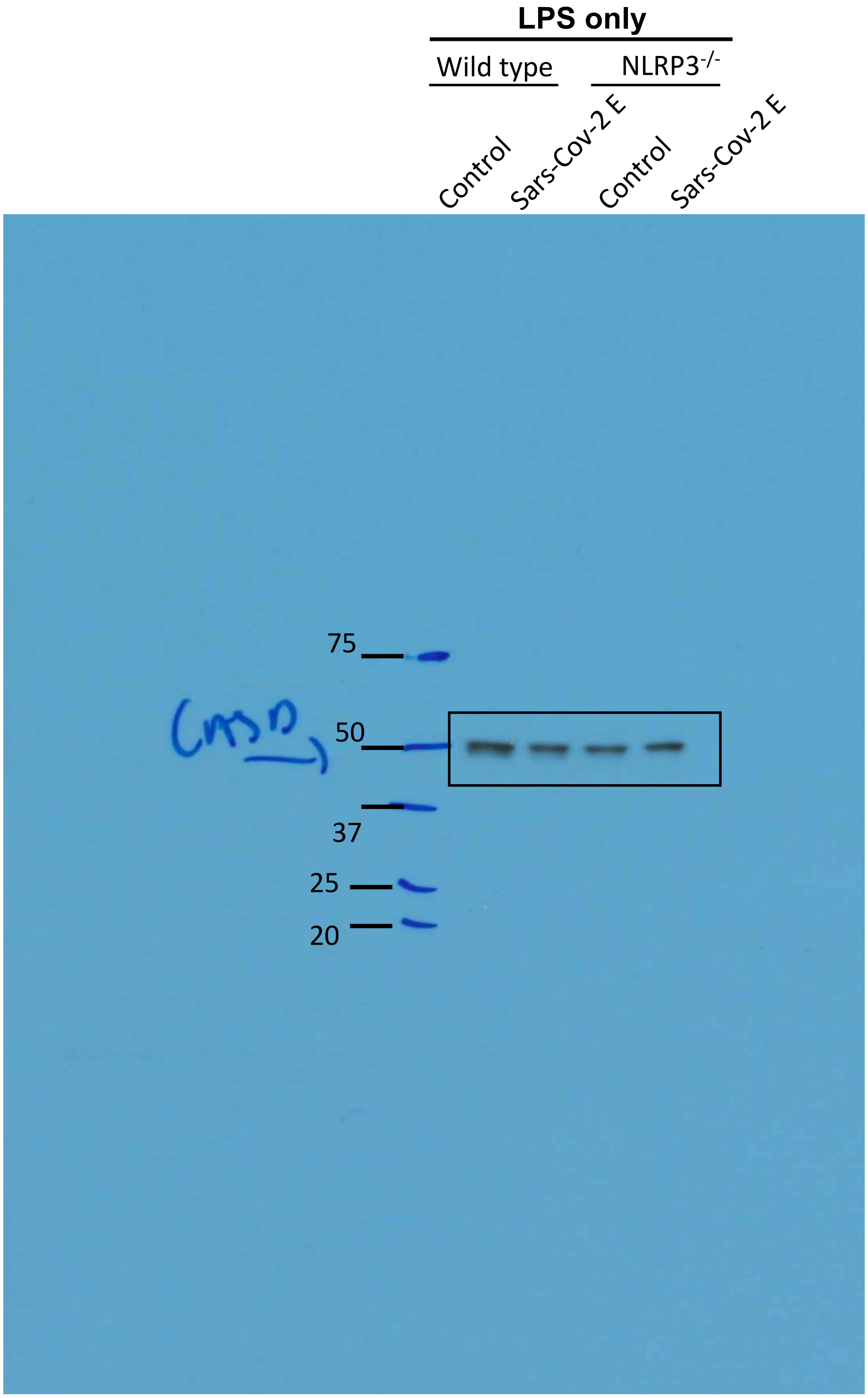
LPS only
Wild type
NLRP3-/-
Control
Sars-Cov-2 E
Control
Sars-Cov-2 E
75
50
37
25
20

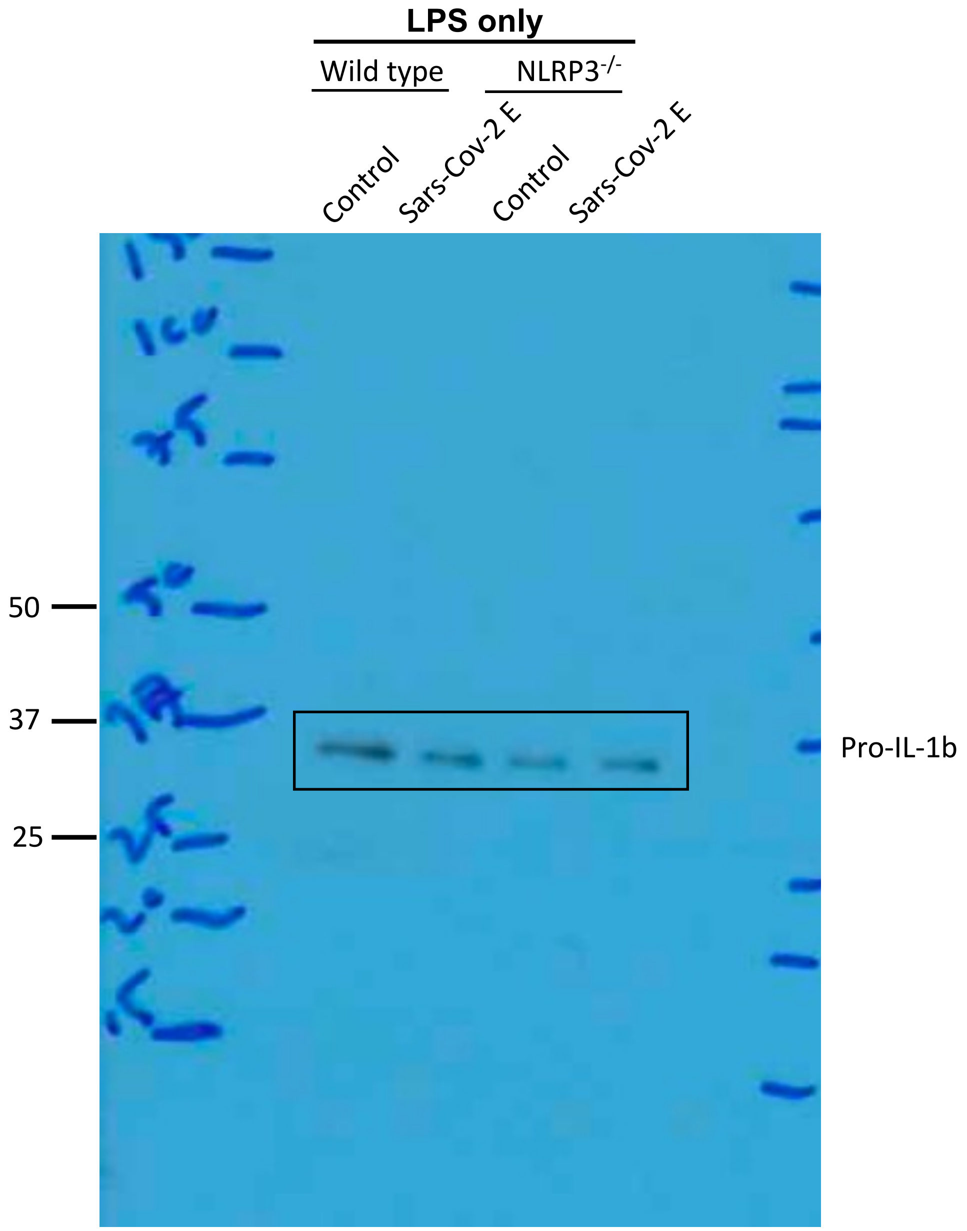
LPS only
Wild type
NLRP3-/-
Control
Sars-Cov-2 E
Control
Sars-Cov-2 E
50
37
25
Pro-IL-1b

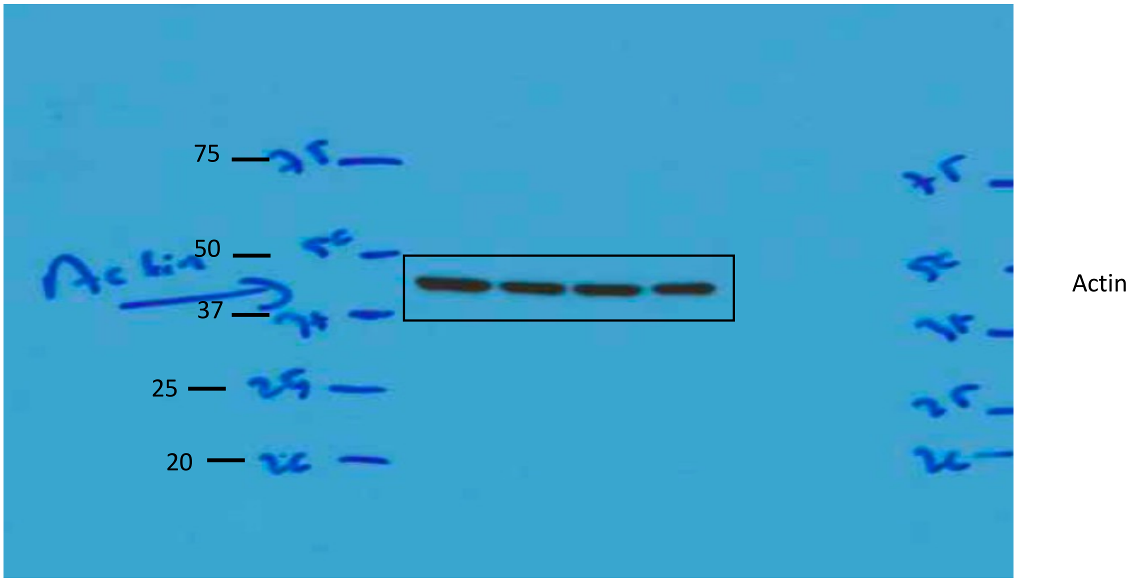

Source Data 1D

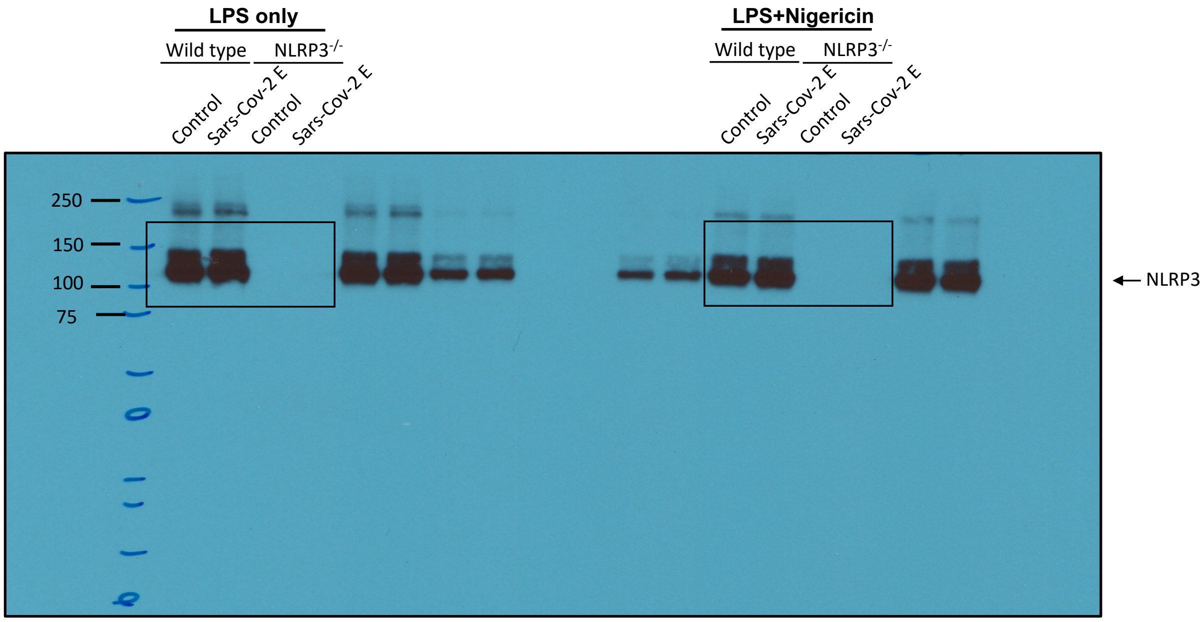

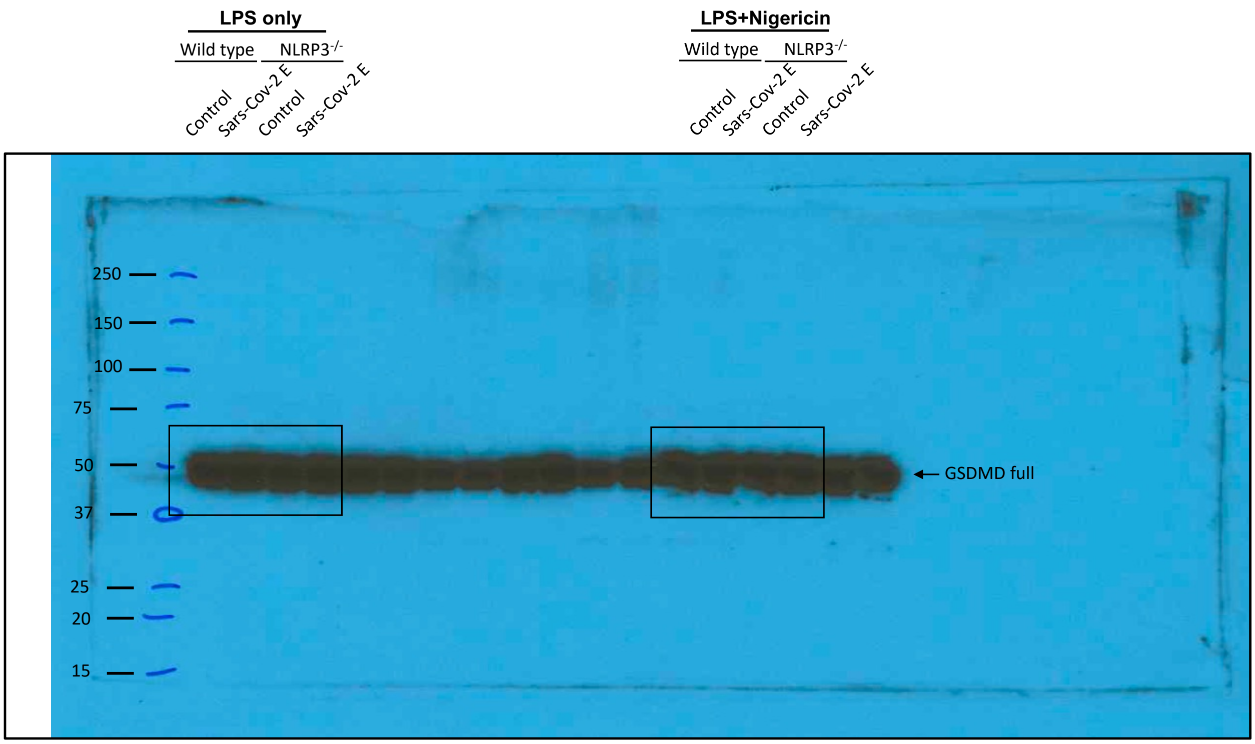
LPS only
Wild type
NLRP3-/-
Control
Sars-Cov-2 E
Control
Sars-Cov-2 E
LPS+Nigericin
Wild type
NLRP3-/-
Control
Sars-Cov-2 E
Control
Sars-Cov-2 E
250
150
100
75
50
37
25
20
15
GSDMD full

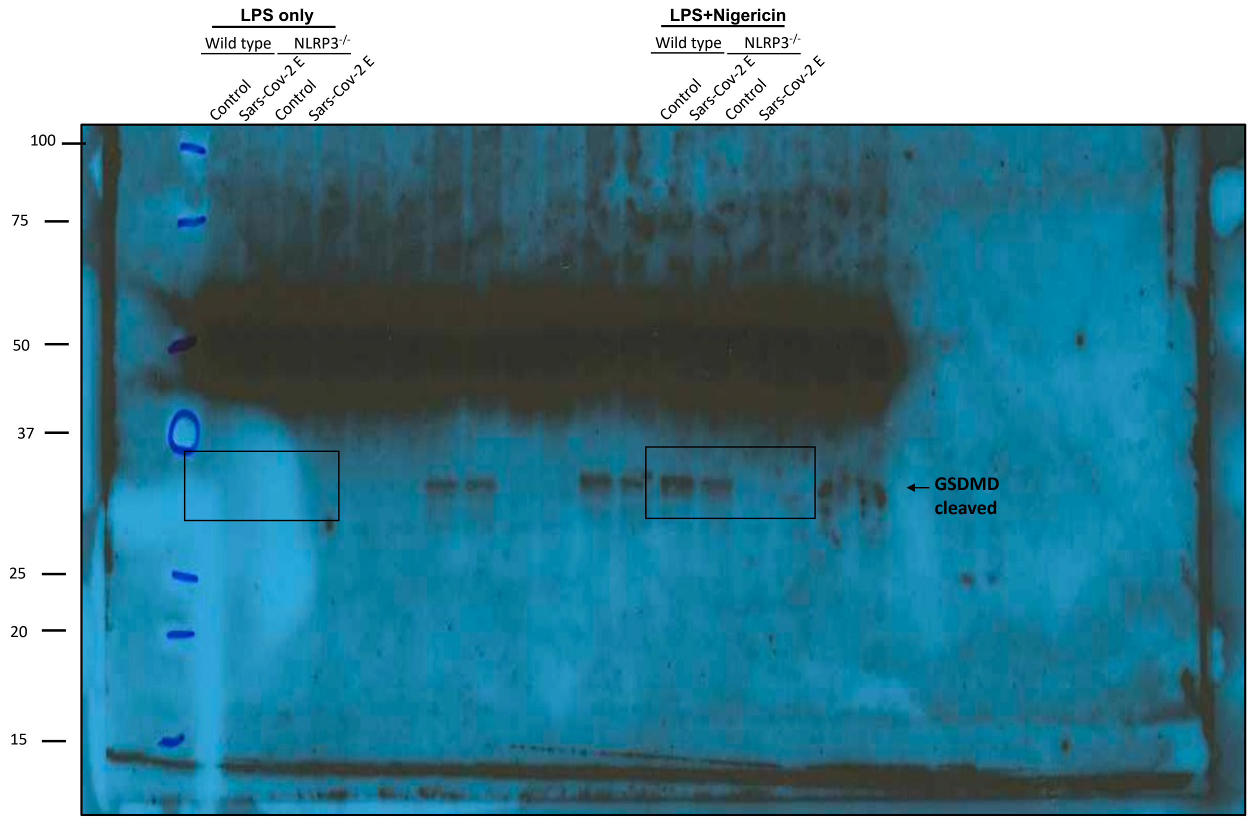
LPS only
Wild type
NLRP3-/-
Control
Sars-Cov-2 E
Control
Sars-Cov-2 E
LPS+Nigericin
Wild type
NLRP3-/-
Control
Sars-Cov-2 E
Control
Sars-Cov-2 E
100
75
50
37
25
20
15
GSDMD
cleaved

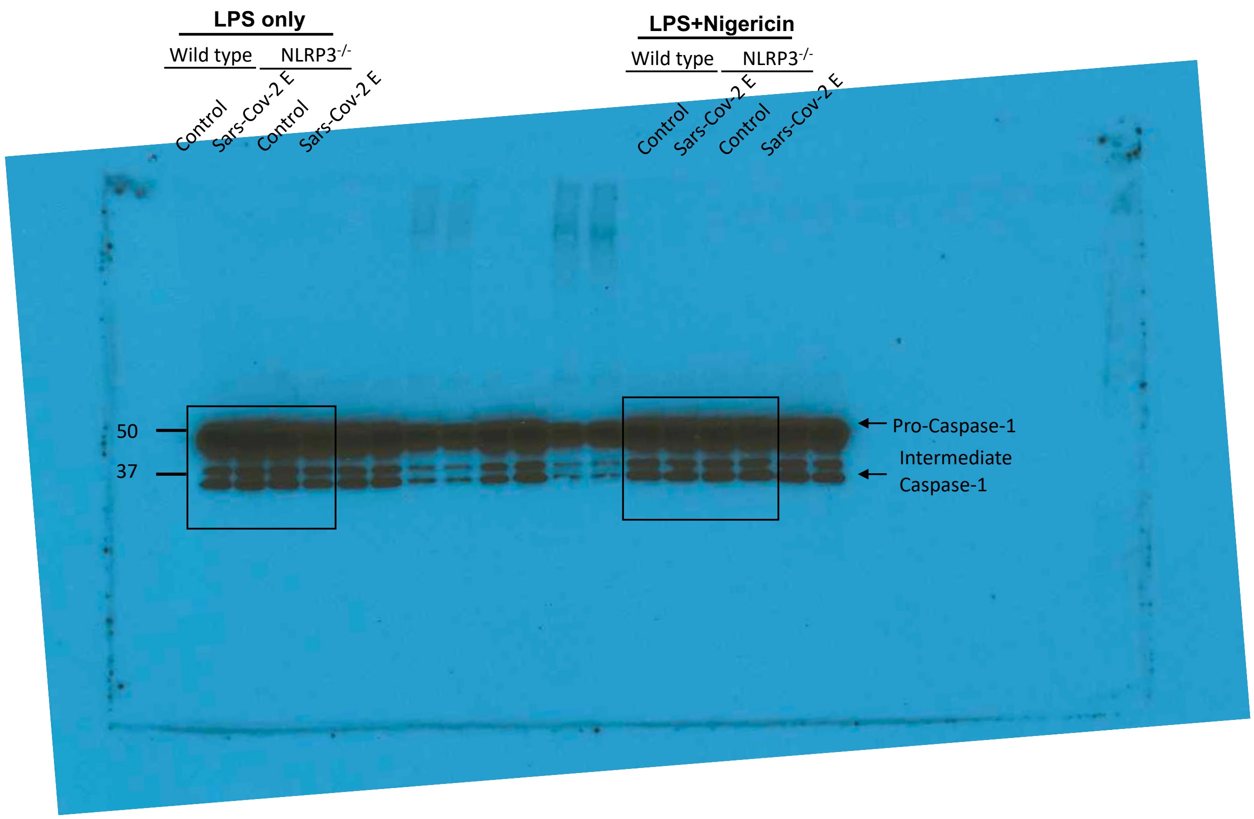
LPS only
Wild type
NLRP3-/-
Control
Sars-Cov-2 E
Control
Sars-Cov-2 E
LPS+Nigericin
Wild type
NLRP3-/-
Control
Sars-Cov-2 E
Control
Sars-Cov-2 E
50
37
Pro-Caspase-1
Intermediate Caspase-1

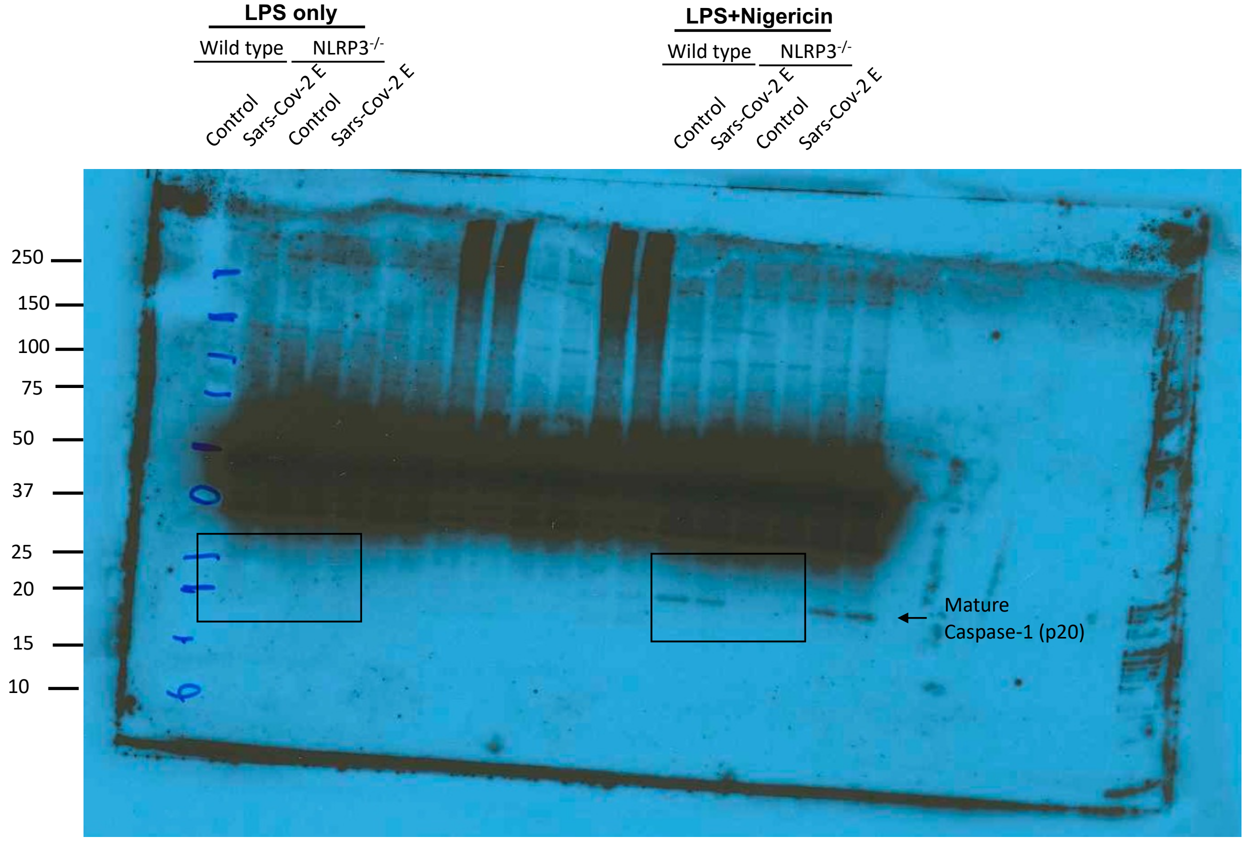
LPS only
Wild type
NLRP3-/-
Control
Sars-Cov-2 E
Control
Sars-Cov-2 E
LPS+Nigericin
Wild type
NLRP3-/-
Control
Sars-Cov-2 E
Control
Sars-Cov-2 E
250
150
100
75
50
37
25
20
15
10
Mature Caspase-1 (p20)

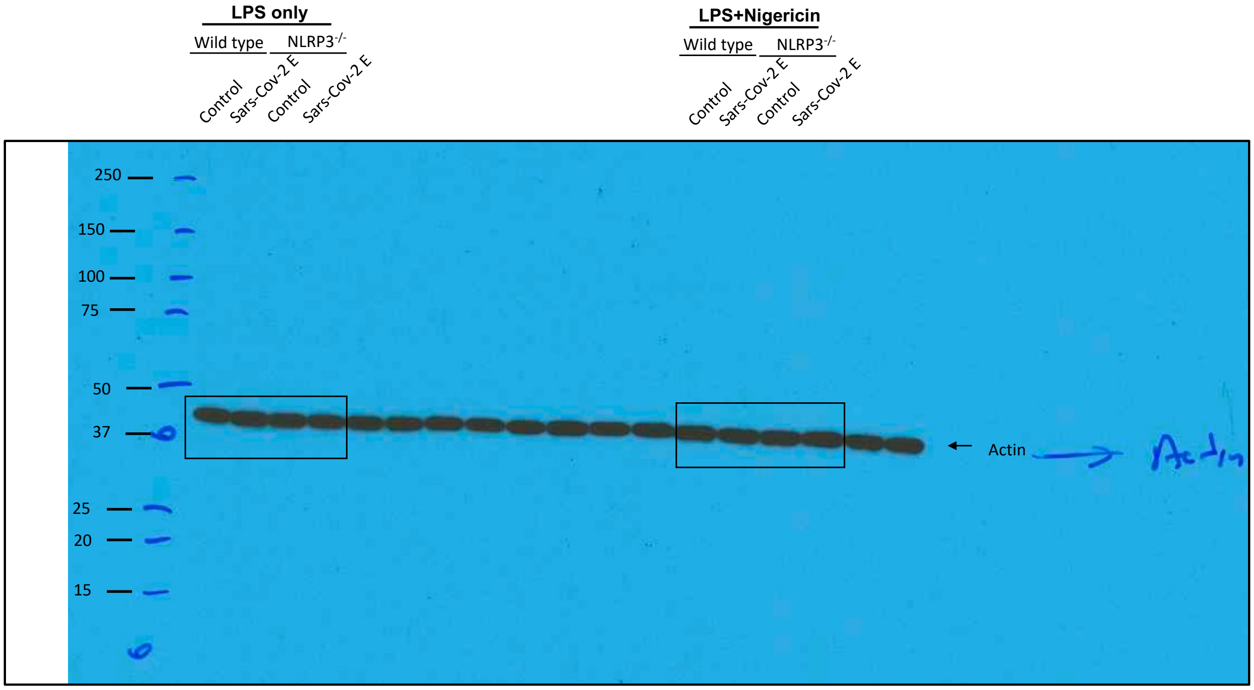
LPS only
Wild type
NLRP3-/-
Control
Sars-Cov-2 E
Control
Sars-Cov-2 E
LPS+Nigericin
Wild type
NLRP3-/-
Control
Sars-Cov-2 E
Control
Sars-Cov-2 E
250
150
100
75
50
37
25
20
15
Actin

Source Data 4A

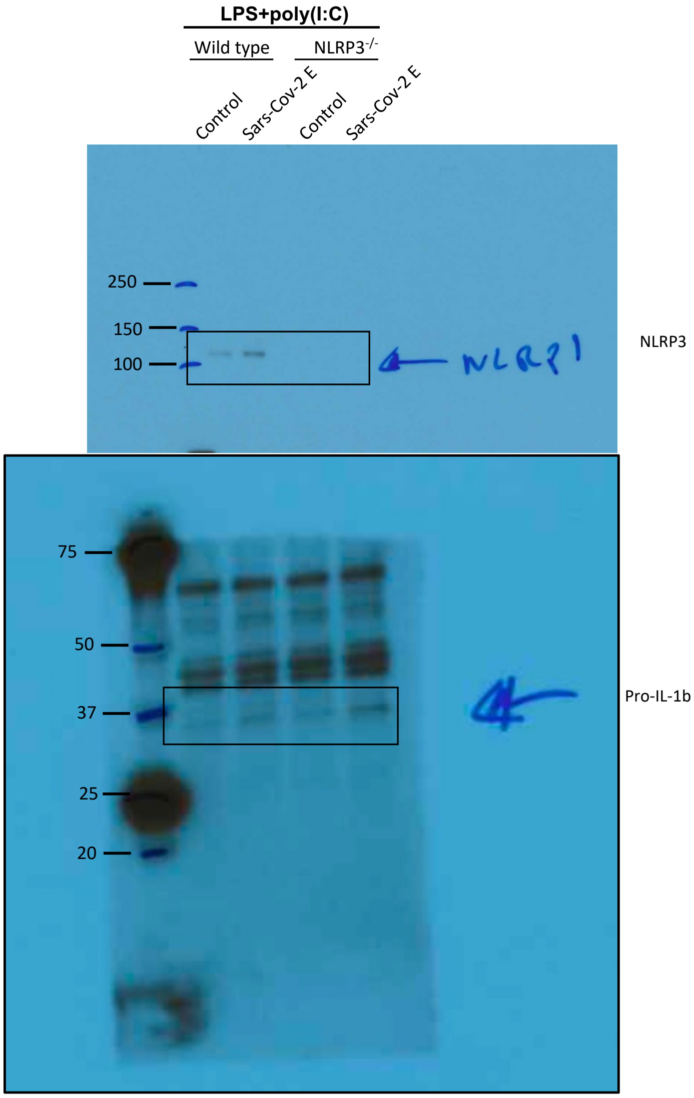

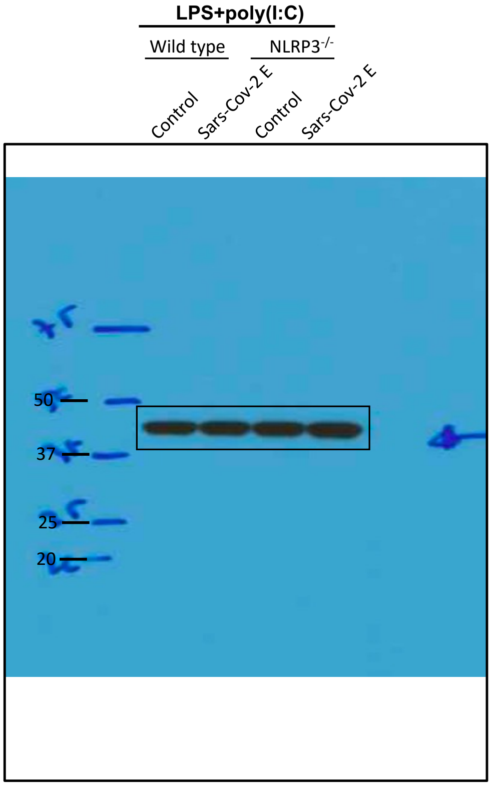

Source Data 2F

Blot 1

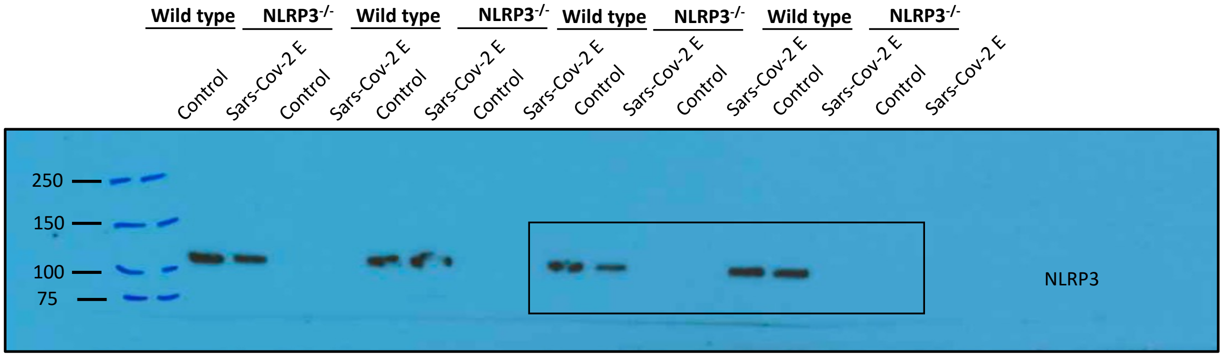

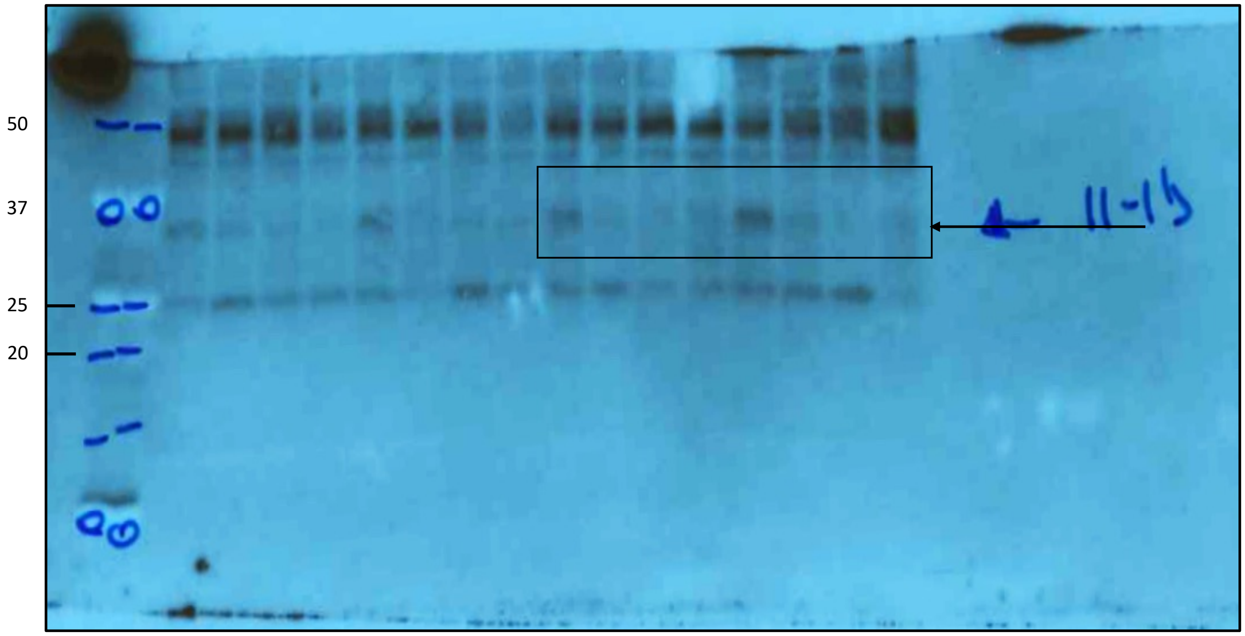

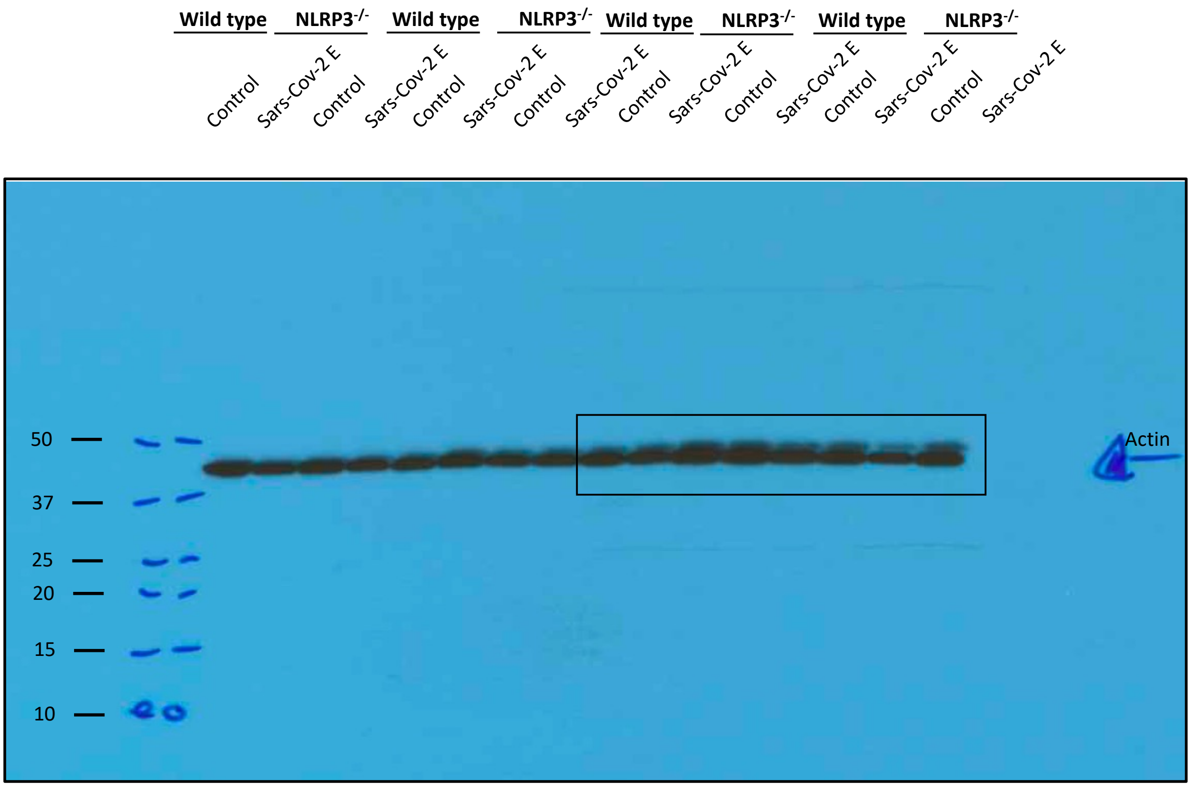
Wild type
NLRP3-/-
Wild type
NLRP3-/-
Wild type
NLRP3-/-
Wild type
NLRP3-/-
Control
Sars-Cov-2 E
Control
Sars-Cov-2 E
Control
Sars-Cov-2 E
Control
Sars-Cov-2 E
Control
Sars-Cov-2 E
Control
Sars-Cov-2 E
Control
Sars-Cov-2 E
Control
Sars-Cov-2 E
50
37
25
20
15
10
Actin

Blot 2

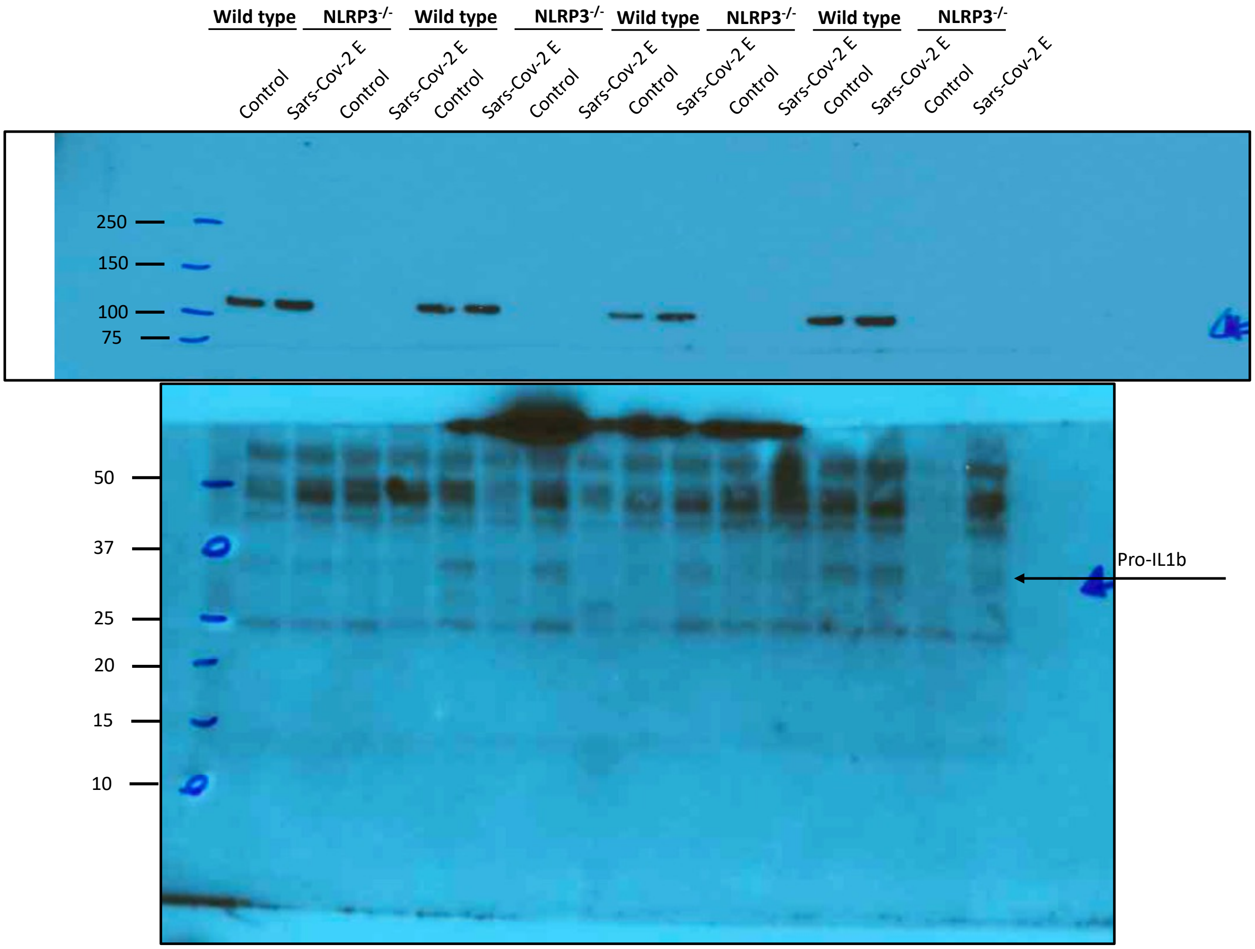
Wild type
NLRP3-/-
Wild type
NLRP3-/-
Wild type
NLRP3-/-
Wild type
NLRP3-/-
Control
Sars-Cov-2 E
Control
Sars-Cov-2 E
Control
Sars-Cov-2 E
Control
Sars-Cov-2 E
Control
Sars-Cov-2 E
Control
Sars-Cov-2 E
Control
Sars-Cov-2 E
Control
Sars-Cov-2 E
250
150
100
75
50
37
25
20
15
10
Pro-IL1b

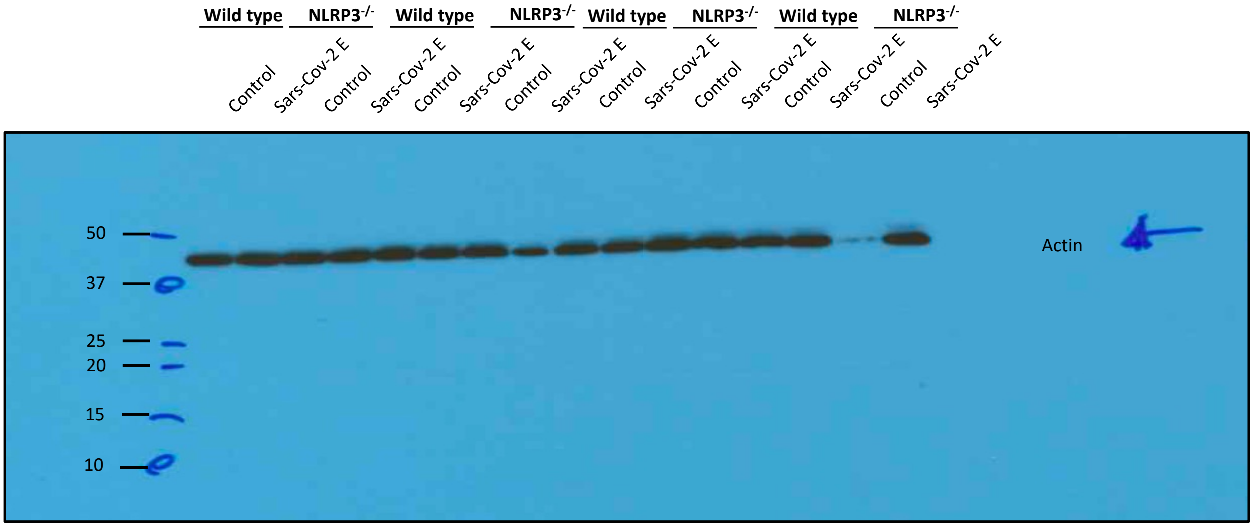
Wild type
NLRP3-/-
Wild type
NLRP3-/-
Wild type
NLRP3-/-
Wild type
NLRP3-/-
Control
Sars-Cov-2 E
Control
Sars-Cov-2 E
Control
Sars-Cov-2 E
Control
Sars-Cov-2 E
Control
Sars-Cov-2 E
Control
Sars-Cov-2 E
Control
Sars-Cov-2 E
Control
Sars-Cov-2 E
50
37
25
20
15
10
Actin

Source Data Supplementary Figure 5B

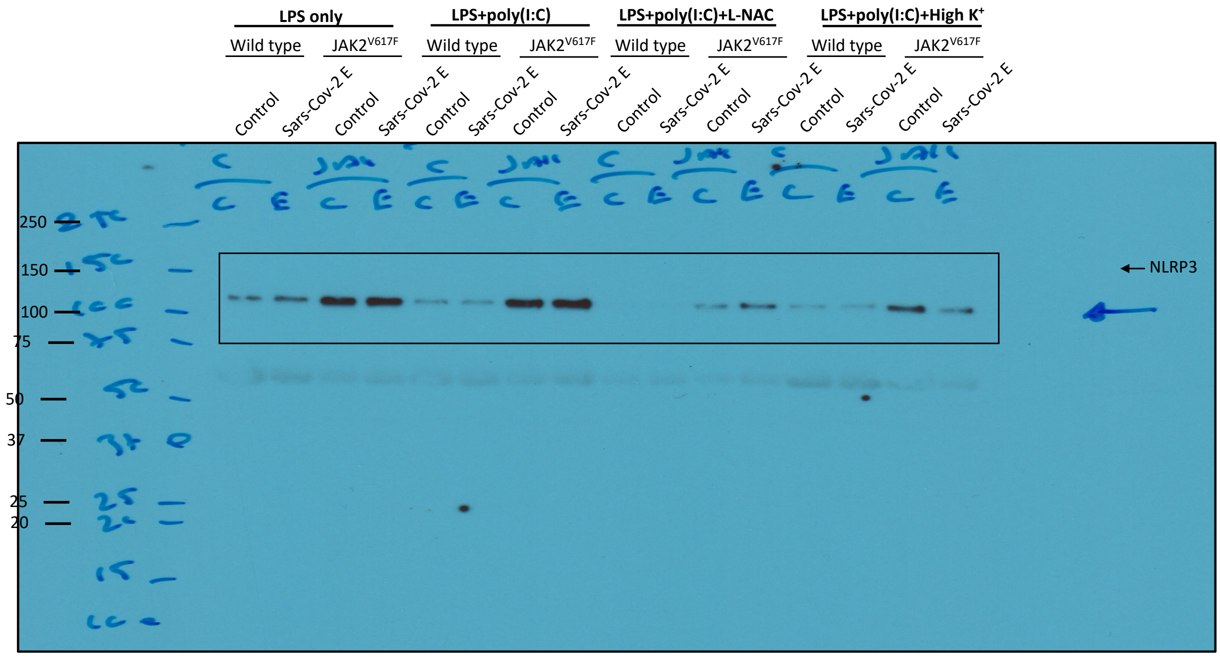

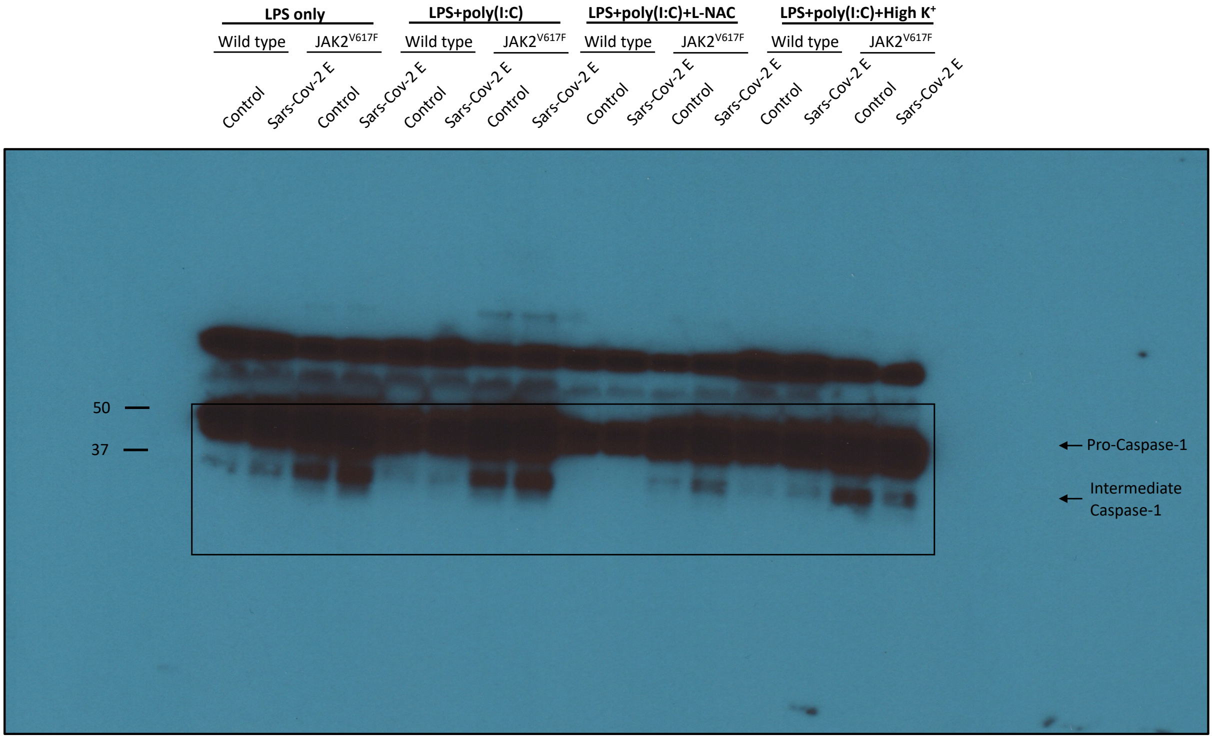

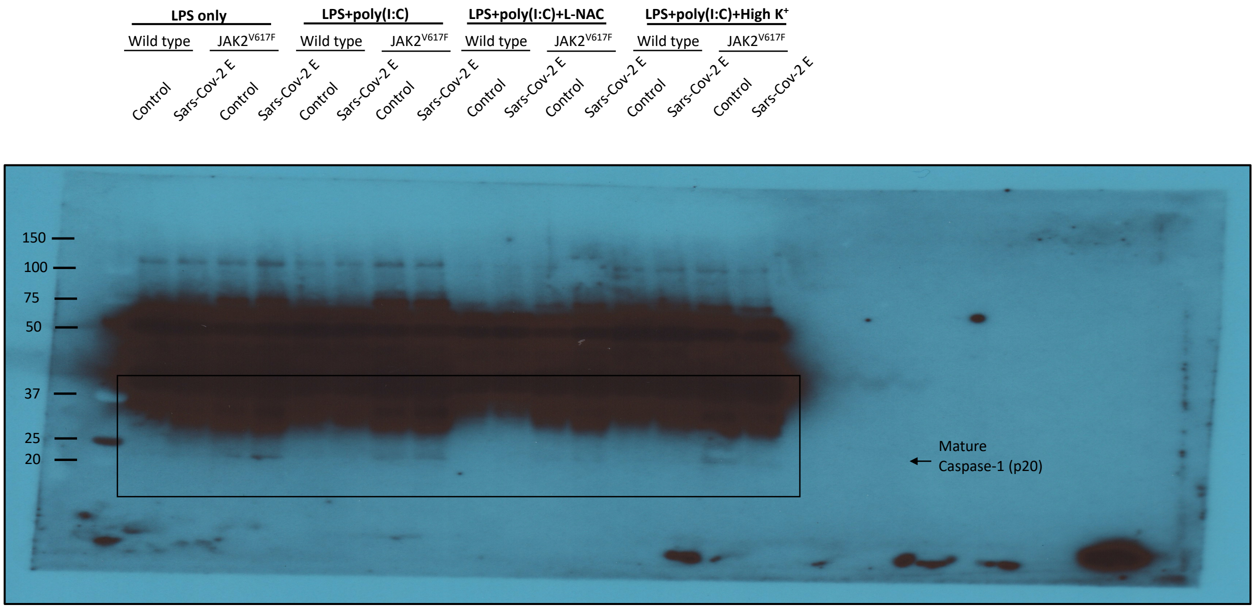
LPS only
Wild type
JAK2V617F
LPS+poly(I:C)
Wild type
JAK2V617F
LPS+poly(I:C)+L-NAC
Wild type
JAK2V617F
LPS+poly(I:C)+High K+
Wild type
JAK2V617F
Control
Sars-Cov-2 E
Control
Sars-Cov-2 E
Control
Sars-Cov-2 E
Control
Sars-Cov-2 E
Control
Sars-Cov-2 E
Control
Sars-Cov-2 E
Control
Sars-Cov-2 E
Control
Sars-Cov-2 E
150
100
75
50
37
25
20
Mature
Caspase-1 (p20)

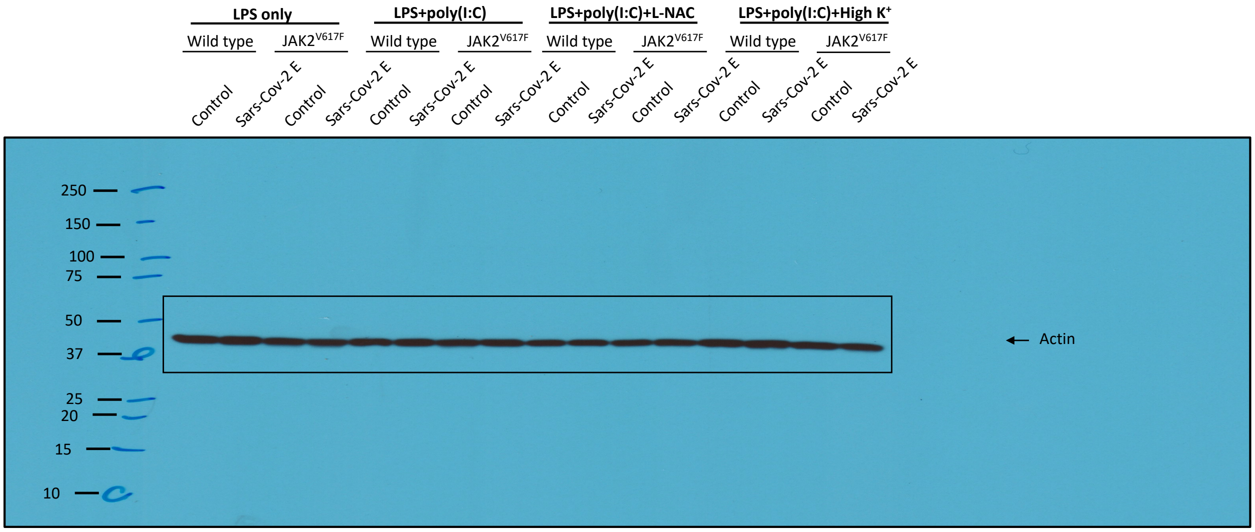
LPS only
Wild type
JAK2V617F
LPS+poly(I:C)
Wild type
JAK2V617F
LPS+poly(I:C)+L-NAC
Wild type
JAK2V617F
LPS+poly(I:C)+High K+
Wild type
JAK2V617F
Control
Sars-Cov-2 E
Control
Sars-Cov-2 E
Control
Sars-Cov-2 E
Control
Sars-Cov-2 E
Control
Sars-Cov-2 E
Control
Sars-Cov-2 E
Control
Sars-Cov-2 E
Control
Sars-Cov-2 E
250
150
100
75
50
37
25
20
15
10
Actin
